# *De Novo* Assembly of *Plasmodium knowlesi* Genomes From Clinical Samples Explains the Counterintuitive Intrachromosomal Organization of Variant *SICAvar* and *kir* Multiple Gene Family Members

**DOI:** 10.3389/fgene.2022.855052

**Published:** 2022-05-23

**Authors:** Damilola R. Oresegun, Peter Thorpe, Ernest Diez Benavente, Susana Campino, Fauzi Muh, Robert William Moon, Taane Gregory Clark, Janet Cox-Singh

**Affiliations:** ^1^ Division of Infection and Global Health, School of Medicine, University of St Andrews, Scotland, United Kingdom; ^2^ Faculty of Infectious and Tropical Diseases, London School of Hygiene & Tropical Medicine, London, United Kingdom; ^3^ Faculty of Epidemiology and Population Health, London School of Hygiene & Tropical Medicine, London, United Kingdom

**Keywords:** *Plasmodium knowlesi*, genomes, clinical samples, *SICAvar*, *kir*, Nanopore, *de novo*, malaria

## Abstract

*Plasmodium knowlesi*, a malaria parasite of Old World macaque monkeys, is used extensively to model *Plasmodium* biology. Recently, *P. knowlesi* was found in the human population of Southeast Asia, particularly Malaysia. *P. knowlesi* causes uncomplicated to severe and fatal malaria in the human host with features in common with the more prevalent and virulent malaria caused by *Plasmodium falciparum*. As such, *P. knowlesi* presents a unique opportunity to develop experimental translational model systems for malaria pathophysiology informed by clinical data from same-species human infections. Experimental lines of *P. knowlesi* represent well-characterized genetically stable parasites, and to maximize their utility as a backdrop for understanding malaria pathophysiology, genetically diverse contemporary clinical isolates, essentially wild-type, require comparable characterization. The Oxford Nanopore PCR-free long-read sequencing platform was used to sequence and *de novo* assemble *P. knowlesi* genomes from frozen clinical samples. The sequencing platform and assembly pipelines were designed to facilitate capturing data and describing, for the first time, *P. knowlesi schizont-infected cell agglutination* (*SICA*) *var* and *Knowlesi-Interspersed Repeats* (*kir*) multiple gene families in parasites acquired from nature. The *SICAvar* gene family members code for antigenically variant proteins analogous to the virulence-associated *P. falciparum* erythrocyte membrane protein (*PfEMP1*) multiple *var* gene family. Evidence presented here suggests that the *SICAvar* family members have arisen through a process of gene duplication, selection pressure, and variation. Highly evolving genes including *PfEMP1*family members tend to be restricted to relatively unstable sub-telomeric regions that drive change with core genes protected in genetically stable intrachromosomal locations. The comparable *SICAvar* and *kir* gene family members are counter-intuitively located across chromosomes. Here, we demonstrate that, in contrast to conserved core genes, *SICAvar* and *kir* genes occupy otherwise gene-sparse chromosomal locations that accommodate rapid evolution and change. The novel methods presented here offer the malaria research community not only new tools to generate comprehensive genome sequence data from small clinical samples but also new insight into the complexity of clinically important real-world parasites.

## Introduction

*Plasmodium knowlesi* is a malaria parasite first described in a natural host, the long-tailed macaque monkey (*Macaca fascicularis*), in the early part of the 20^th^ century (Knowles and Gupta, 1932). Although an incidental find, *P. knowlesi* was soon exploited as a model parasite for malaria research as recently reviewed (Butcher and Mitchell, 2018; Galinski et al., 2018; Pasini et al., 2018). Experimental *P. knowlesi* was well characterized over time with several lines adapted from natural macaque hosts and one human infection originating in geographically distinct regions (Chin et al., 1965; Chin et al., 1968; Butcher and Mitchell, 2018; Galinski et al., 2018; Pasini et al., 2018). Taken together, experimental lines of *P. knowlesi* remain important members of the malaria research arsenal.

What sets *P. knowlesi* apart is that it occupies several important niche areas—as an experimental model, a natural parasite of Southeast Asian macaque monkeys, and the causative agent of zoonotic malaria in the human host (Singh et al., 2004). In nature, transmission is established in the jungles of Southeast Asia, areas that support the sylvan mosquito vectors, the parasite, and the natural macaque hosts. People who enter transmission zones are susceptible to infected mosquito bites and infection. *P. knowlesi* has effectively crossed the vertebrate host species divide and is responsible for malaria in contemporary human hosts (World-Health-Organization, 2021).

Zoonotic malaria caused by *P. knowlesi* is currently the most common type of malaria in Malaysia, with most of the cases reported in Malaysian Borneo (Chin et al., 2020). Indeed, naturally acquired *P. knowlesi* malaria causes a spectrum of disease from uncomplicated to severe and fatal infections with tantalizing similarity to severe adult malaria caused by *P. falciparum* (Cox-Singh et al., 2008; Daneshvar et al., 2009; Cox-Singh et al., 2010; Daneshvar et al., 2018).

The clinical similarities observed in patients with severe *P. knowlesi* and *P. falciparum* infections suggest that *P. knowlesi* has the potential to serve as a translational animal model system for severe malaria pathophysiology that has hitherto eluded medical science (Ozwara et al., 2003; Cox-Singh et al., 2010; Cox-Singh and Culleton, 2015; Onditi et al., 2015; Cox-Singh, 2018).

To take this idea forward, it seemed prudent to compare genome sequences derived from contemporary clinical isolates of *P. knowlesi* with *P. knowlesi* genomes generated from *P. knowlesi*-infected red blood cells propagated in rhesus monkeys (Pain et al., 2008; Lapp et al., 2018) or *in vitro* cultures (Benavente et al., 2018). Our data from clinical isolates were primarily compared to the first reference genome (Pain et al., 2008) and a cultured parasite (PkA1-H.1) (Benavente et al., 2018) control reference sequence and assembly generated using the same procedures.

Previously, we developed methods to produce high-quality Illumina short-read *P. knowlesi* genome sequence data from frozen clinical blood samples (Pinheiro et al., 2015). The outputs of that work identified genome-wide diversity, including a genomic dimorphism in *P. knowlesi* isolated from patients, but the Illumina platform was not suitable to resolve complex multiple gene family members.

*Plasmodium* species have a number of multiple gene families that code for infected host red blood cell surface proteins. The proteins are antigenic and highly variable to avoid host immune recognition and parasite destruction (Wahlgren et al., 2017; Harrison et al., 2020). Among these are the *P. falciparum* erythrocyte membrane protein (*PfEMP1*) gene family members with an estimated 67 copies in the *P. falciparum* 3D7 reference genome and variable copy numbers in clinical isolates (*n* = 47–90) (Gardner et al., 2002; Otto et al., 2018). *PfEMP1* genes are expressed in a mutually exclusive manner with only one predominantly expressed at any one time (Hviid and Jensen, 2015; Abdi et al., 2017; Andrade et al., 2020). Importantly, *PfEMP1* gene expression is implicated in *P. falciparum* virulence and progression to severe disease (Lavstsen et al., 2012; Abdi et al., 2017; Shabani et al., 2017; Wahlgren et al., 2017; Milner, 2018; Tessema et al., 2019; Jensen et al., 2020). While other multiple gene families are described in all *Plasmodium* species studied to date, *PfEMP1* gene-like families are rare, and among the parasites that cause human disease, they are found only in *P. falciparum* and *P. knowlesi* (Gardner et al., 2002; Pain et al., 2008). The comparable *P. knowlesi schizont-infected cell agglutination variant antigen* (*SICAvar*) gene family has been reported in detail in experimental parasites that had been passaged in rhesus monkeys (al-Khedery et al., 1999; Pain et al., 2008; Lapp et al., 2015; Galinski et al., 2018; Lapp et al., 2018) but to our knowledge not in wild-type parasites, including *P. knowlesi* isolated from patients. Given the *PfEMP1* gene association with severe disease in *P. falciparum,* we are particularly interested in describing *P. knowlesi SICAvar* multiple gene family member organization, location, and copy number in clinical isolates using amplification-free genome sequencing. With the methodology presented here and in the study by Oresegun et al. (2021), we can move forward in subsequent studies to achieve this goal.

Multiple gene family members are similar with long stretches of regions of low complexity that require long-read sequencing technologies to resolve (Pinheiro et al., 2015; Heather and Chain, 2016). Recently, the PacBio long-read sequencing platform was used to describe, for the first time, the core *P. falciparum* genome in clinical isolates and demark sub-telomeric regions to compare genome organization and diversity between clinical isolates from different geographical regions and the commonly used *P. falciparum* clone 3D7 (Otto et al., 2018).

The PacBio platform is outside of our reach because we have small-volume frozen whole blood samples that yield parasite DNA well below the quantity required for amplification-free PacBio sequencing (Benavente et al., 2018; Lapp et al., 2018; Otto et al., 2018). Here, we use the accessible, portable, and affordable Oxford Nanopore Technologies MinION long-read sequencing platform, suitable for small-quantity input DNA, to sequence and *de novo* assemble two new *P. knowlesi* reference genome sequences representing each genetically dimorphic form of *P. knowlesi* found in our patient cohort (Pinheiro et al., 2015; Ahmed et al., 2014).

The new reference genomes will, for the first time, provide insight into clinically relevant contemporary *P. knowlesi* parasites. These diverse parasites are essentially wild-type and the product of ongoing mosquito transmission and recombination in nature (Assefa et al., 2015; Pinheiro et al., 2015; Divis et al., 2018; Ahmed and Quan, 2019; Fong et al., 2019). The genomes will offer a valuable resource not only for our studies on members of the *SICAvar* gene family and virulence but also to the wider malaria research community working on comparative biology of malaria parasites, drug discovery, and vaccine development.

## Materials and Methods

### Sample Selection

*P. knowlesi* DNA extracted from archived clinical samples collected with informed consent as part of a non-interventional study were used (Ahmed et al., 2014). The isolates were selected to represent each of the two genetically distinct clusters, KH273 (sks047) and KH195 (sks048), of *P. knowlesi*–infected patients in the study cohort (Ahmed et al., 2014; Pinheiro et al., 2015). Control *P. knowlesi* DNA was extracted from the experimental line *P. knowlesi* A1-H.1 adapted to *in vitro* culture in human erythrocytes, the culture kindly donated by Robert Moon (Moon et al., 2013). In order to distinguish the genome data generated here for *P. knowlesi* A1-H.1 from those already existing, we use the unique abbreviation StAPkA1H1 (Diez Benavente et al., 2017; Benavente et al., 2018).

### Plasmodium DNA Extraction

Human DNA was depleted from 200 to 400 µl thawed clinical samples using a previously described method (Oresegun et al., 2021). Briefly, surviving human leucocytes in thawed samples were removed using anti-human CD45 DynaBeads (ThermoFisher Scientific). The resulting parasite pellet was washed to remove soluble human DNA (hDNA), and parasite-enriched DNA (pDNA) was extracted using the QIAamp Blood Mini Kit (QIAGEN) with final elution into 150 µl AE buffer. DNA concentrations were quantified using the Qubit 2.0 fluorometer (Qubit™, Invitrogen) and real-time qPCR on RotorGene (QIAGEN). Recovered DNA was concentrated, and short fragments were removed by mixing at a ratio of 1:1 by volume with AMPureXP magnetic beads (Beckman Coulter) following the manufacturer’s instructions. Briefly, the AMPureXP bead mixture was placed in a magnetic field, and DNA bound to the beads was rinsed twice with 70% ethanol before air-drying to allow residual ethanol to evaporate. Parasite-enriched DNA was eluted in 10 µl nuclease-free H_2_O (Ambion). One microliter of recovered DNA concentrate was used for DNA quantification using a Qubit fluorimeter (ThermoFisher Scientific), and 7.5 µl was taken forward for sequencing library preparation.

### Library Preparation and Sequencing

Parasite-enriched DNA was sequenced using the Oxford Nanopore Technologies (ONT) MinION long-read sequencing platform. Library preparations were selected to suit PCR-free sequencing for the small pDNA quantities available to study (∼400 ng). Sequencing libraries were prepared following the manufacturer’s instructions for the SQK-RBK004 ONT sequencing kit. Sequencing was performed using R9.4.1 flowcells or R10 flowcells (Oresegun et al., 2021). Previously sequenced Illumina reads for the patient isolates (sks047 and sks048) were retrieved from the European Nucleotide Archive, with accession codes ERR366425 and ERR274221, respectively (Pinheiro et al., 2015). Further short-read sequencing was carried out on PCR-enriched DNA using the Illumina MiSeq platform at the London School of Hygiene and Tropical Medicine and methods established by Diez Benavente et al. (2019).

### Reference Genomes

For chromosome scaffolding and quality assessment comparison, the *P. knowlesi* PKNH reference genome (Pain et al., 2008) (version 2) was downloaded from Sanger (ftp://ftp.sanger.ac.uk/pub/genedb/releases/latest/Pknowlesi/#). In addition, further comparisons were carried out using the *P. knowlesi* PkA1H1 reference genome (Benavente et al., 2018) from NCBI [accession code: GCA_900162085].

### *De Novo* Genome Assembly

MinION FAST5 file outputs were locally base called using the high accuracy model of the guppy basecaller (v4.0.15; Ubuntu 19.10; GTX1060) with the following parameters: “*-r -v -q 0 --qscore-filtering -x auto*.” Demultiplexing was carried out using qcat software (v1.1.0) with the “*--detect-middle --trim -k --guppy*” parameters, and then adapter removal was carried out using porechop (v0.2.4) with default parameters and the most recent versions released from ONT technologies. Human DNA (hDNA) contamination was removed from the adapter-free reads by alignment against the human GRCh38.p13 reference genome (retrieved from NCBI accession code: GCF_000001405.39) (Lander et al., 2001) using minimap2 (v2.17) (Li, 2018) with “*-ax map-ont*” default parameters. Unmapped reads were separated from the binary sequence alignment (BAM) file using samtools (v1.10) (Li et al., 2009; Li, 2011) and converted back to FASTQ using bedtools (v2.29.2) (Quinlan and Hall, 2010) for *de novo* genome assembly using Flye (v2.8.1) (Kolmogorov et al., 2019) with an expected genome size of 25 Mb and “*--nano-raw*” default parameters. Successful assemblies were assessed for contamination using BlobTools (v1.0.1) (Laetsch and Blaxter, 2017). Contigs not taxonomically assigned as Apicomplexan were discarded.

### Assembly Polishing and Correction

Draft assemblies were polished using four iterations of racon (v1.4.13) (Vaser et al., 2017); in the default setting, raw long-read isolate sequence reads which did not align to the human GRCh38.p13 (henceforth parasite-reads) were retained. As part of the polishing step, alignments of parasite-reads against the draft assembly were performed using minimap2 (v2.17) (Li, 2018). A consensus sequence was subsequently generated from the racon output using medaka (v1.0.3; default settings) (Oxford Nanopore Technologies, 2019). Further polishing and correction were carried out using Illumina paired-end reads where available, using three iterations of pilon (v1.23) with default parameters “*-Xmx120G, --tracks, --fix all, circles*” (Walker et al., 2014).

### Masking Repetitive Elements

The *P. knowlesi* PKNH reference mitochondrial (MIT) and apicoplast (API) sequences were extracted and individually aligned against draft *P. knowlesi* assemblies using MegaBLAST (v.2.9; default parameters) (Morgulis et al., 2008; Pain et al., 2008). Contigs which aligned to the reference PKNH MIT and API genomes were subsequently removed and circularized on Circlator (v1.5.5) (Hunt et al., 2015) with the command “*circlator all --data_type nanopore-raw --bwa_opts ‘-x ont2d’ --merge_min_id 85 --merge_breaklen 1000*.” API/MIT-free draft nuclear assemblies (henceforth, draft assemblies) were taken forward through RepeatModeler (v1.0.10) (Flynn et al., 2020), and the outputs were utilized as input for Censor (Kohany et al., 2006) where the options “*Eukaryota*” and “*Report simple repeats*” were selected. Identified transposable elements and repeats in the censor outputs were classified based on the class of repeats to make a repeat library for each assembly. Repeat libraries of each draft assembly were combined and misplaced, redundant sequences were removed using CD-HIT (v4.8.1; “*-c 1.0 -n 10 -d 0 -g 1 -M 60000*” parameters) (Li and Godzik, 2006; Fu et al., 2012). This generated a singular “master” repeat library encompassing the non-redundant list of identified elements across the three draft assemblies.

With the master repeat library, RepeatMasker (v4.0.7) was run on each draft assembly producing a tab-separated value (TSV) output of the identified repeats in the assembly. Then, using ‘One Code to Find Them All’ (OCFTA) (Bailly-Bechet et al., 2014), each TSV file was parsed to clarify further repeat positions found using RepeatMasker. Next, the LTRHarvest (Ellinghaus et al., 2008) module of GenomeTools (v1.6.1) (Gremme et al., 2013) was used to find secondary structures of long terminal repeats (LTRs) and other alternatives in the DRAFT assemblies. Here, the “*suffixerator*” function was implemented with “*-tis -suf -lcp -des -ssp -sds -dna*” parameters while the “*ltrharvest*” function was run with “*-mintsd 5 -maxtsd 100*”’ parameters. Concurrently, TransposonPSI was also used on the DRAFT assemblies with default parameters to find repeat elements based on their coding sequences.

Redundant repeat element sequences were removed from the outputs of RepeatMasker, OCTFA, LTRHarvest, and TransposonPSI using a custom script, to generate a genome feature file (GFF3) where each transposable and repetitive element of each DRAFT assembly is represented once. Then, within each draft assembly, repeat elements were masked using the coordinates present in the non-redundant GFF3 file and the “*maskfasta*” function of bedtools (v2.27; default settings and “*-soft*”).

### Prediction and Annotation

The masked draft assemblies were checked for chimeric contigs using Ragtag (v1.0.1) (Alonge et al., 2019) where both the “*correct*” and “*scaffold*” functions were run with the “*--debug --aligner nucmer --nucmer-params = ‘-maxmatch -l 100 -c 500’*” parameters (Li et al., 2009; Li, 2011).

With the chimeric contigs broken, masked draft assemblies were uploaded on the Companion webserver (Steinbiss et al., 2016) for gene prediction and annotation using the sequence prefix of “PKA1H1_STAND” for the cultured experimental line (StAPKA1H1) and “PKCLINC” for patient isolates (sks047 and sks048). Companion software was run with no transcript evidence, 500 bp minimum match length, and 80% match similarity for contig placement, 0.8 AUGUSTUS (Stanke et al., 2006) score threshold, and taxid 5851. Additionally, pseudochromosomes were contiguated, reference proteins were aligned to the target sequence, pseudogene detection was carried out, and RATT was used for reference gene models.

### Comparative Genomics, Quality Assessment and Analyses

As the pipeline progressed, assembly metrics were checked using assembly-stats (v1.0.1) and pomoxis (v0.3.4). Additionally, draft genomes were further assessed for completeness and accuracy using Benchmarking Universal Single-Copy Orthologues (*BUSCO*) v5.0 with “*-l plasmodium_odb10 -f -m geno --long*” parameters (Simão et al., 2015). GFF3 files generated on Companion were parsed for genes of interest, including multigene families known to span the core genome and telomeric regions. Chromosomes of the annotated draft genomes were individually aligned against the corresponding *P. knowlesi* PKNH reference chromosome (Pain et al., 2008) with minimap2 parameters “*-ax asm5*. ” The resulting alignment files were analyzed on Qualimap (v.2.2.2) (Okonechnikov et al., 2016) with parameters “*−nw 800−hm 7*. ” Gene density, chromosome structure, and multigene family plots were generated using the karyoploteR visualization package (Gel and Serra, 2017). Dotplots to identify repetitions, breaks, and inversions were generated from minimap2 whole genome alignments using D-GENIES default settings (Cabanettes and Klopp, 2018).

### Structural Variant Analyses

The StAPkA1H1 draft genome, assembled here, was used as the reference for structural variant calling and subsequent variant annotation to ensure parity across sequencing technologies. Read alignment-based structural variant calling (henceforth reads-based) was achieved using the Oxford Nanopore structural variation pipeline (ONTSVP) (https://github.com/nanoporetech/pipeline-structural-variation), while the assembly-based approach was completed with Assemblytics (Nattestad and Schatz, 2016). Using a modified Snakefile, FASTQ isolates parasite-reads and the StAPkA1H1 draft genome; the ONTSVP first parses the input reads using catfishq (https://github.com/philres/catfishq) and seqtk (https://github.com/lh3/seqtk) before carrying out alignment using lra with parameters “*-ONT -p s*” (Ren and Chaisson, 2020). The resulting alignment file was sorted and indexed using samtools, and read coverage was then calculated using mosdepth (“−*x*−*n*−*b 1000000*”) (Pedersen and Quinlan, 2018). Structural variants (SVs) were called using cuteSV (Jiang et al., 2020) with parameters “*--min-size 30 --max-size 100,000 --retain_work_dir --report_readid --min_support 2*.” Variants were subsequently filtered for length (30 bp), depth (8 reads), quality (Q30), and structural variant type (SVTYPE) such as insertions (INS) by default, before filtered variants were sorted and indexed. Failed SV types were manually filtered based on length (30 bp) and quality (Q30) alone to determine the presence of high-quality, low-occurrence variants.

For the assembly-based structural variant calling for the clinical isolates sks047 and sks048 and StAPkA1H1, draft genomes were aligned against the PKNH reference genome (Pain et al., 2008) using nucmer with “--maxmatch *-l 100 -c 500*” parameters and outputs uploaded onto Assemblytics (http://assemblytics.com) (Nattestad and Schatz, 2016) with default parameters and a minimum SV length of 30 bp. BEDfile outputs of Assemblytics were converted to variant call format (VCF) files using SURVIVOR (v1.0.7) (Jeffares et al., 2017). VCF files for successful reads-based and assembly-based SV calling as well as the failed SV-type VCF files were further filtered to remove any variants less than 50 bp in length and less than Q5 in quality using a bcftools one-liner (https://github.com/samtools/BCFtools). A quality filter was not applicable for the assembly-based approach due to the lack of quality information in the original BEDfile output of Assemblytics. Variants exceeding these thresholds were annotated using vcfanno (v0.3.2) (Nattestad and Schatz, 2016) and subsequently sorted and indexed. Annotated variants, relevant BAM alignment files, and GFF files were visualized on IGV (Thorvaldsdóttir et al., 2013). Using IGV, a gene locus previously identified to be associated with dimorphism—*PknbpX*a (Pinheiro et al., 2015)—was analyzed to determine the presence of structural variants. Summary statistics were calculated using the ‘stats’ function of SURVIVOR with parameters “−1−1−1.” VCF files were compared using the “isec” function of bcftools with default settings, including analyses of the variants present within genes.

### Duplication, Clustering, Genomic Organization and dN/dS Analyses

Scripts used can be found here: https://github.com/peterthorpe5/plasmidium_genomes. Gene duplication analyses were performed using the similarity searches from DIAMOND-BlastP (1e-5) with the MCSanX toolkit (Wang et al., 2012). Orthologues clustering and dN/dS were performed as described in the study by Thorpe et al. (2018). Briefly, OrthoFinder (v2.2.7) (Emms and Kelly, 2019) was used to cluster all the amino acids sequences for the genomes used in this study. The resulting sequences from the clusters of interest were aligned using MUSCLE (v3.8.1551) (Edgar, 2004) and refined using MUSCLE. The resulting amino acid alignment was used as a template to back-translate the nucleotide coding sequence using Biopython for subsequent nucleotide alignment (Cock et al., 2009). The nucleotide alignment was filtered to remove any insertions and deletions and return an alignment with no gaps using trimAL (v1.4.1) (Capella-Gutierrez et al., 2009). The resulting alignment was subjected to dN/dS analysis using Codonphyml (v1.00 201407.24) (-m GY --fmodel F3X4 -t e -f empirical -w g -a e) (Gil et al., 2013). Genomic organization of classes of genes of interest was performed as described in the studies by Eves-van den Akker et al. (2016) and Thorpe et al. (2018, 2020). For UpSet visualization the scripts can be found in the github link above.

## Results

### Evaluating Draft *de novo* Genomes

**FIGURE 1:**
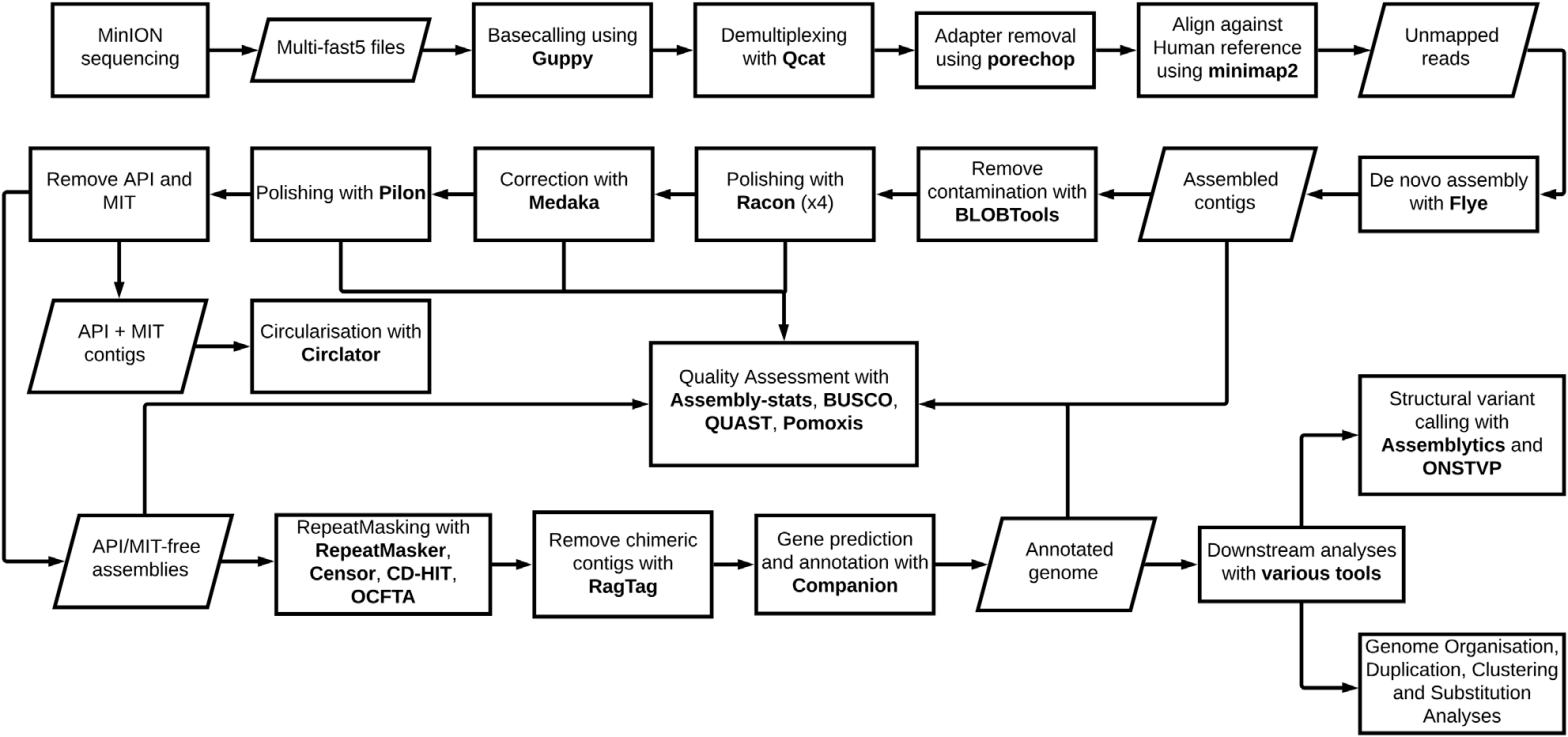
*Plasmodium knowlesi de novo* genome pipeline. The pipeline represents major forms of manipulation taken and tools utilized to generate, annotate, and analyze the two reference genomes derived from clinical isolates and the experimental line.

The genome pipeline, beginning with Oxford Nanopore Technologies (ONT) MinION sequencing through to *de novo* assembly and genome annotation with downstream analyses, is shown (Figure 1). The pipeline was used to produce *P. knowlesi* genomes using DNA extracted from two clinical isolates, sks047 and sks048, and, for comparison, DNA extracted from the well-characterized cultured line, *P. knowlesi* A1-H.1. For the purpose of clarity, the *P. knowlesi* A1-H.1 *de novo* draft genome assembled here is referred to as StAPkA1H1 (please see the Methods section). Read coverages of 225x, 71x, and 65x were obtained for StAPkA1H1, sks047, and sks048, respectively (Table 1). The draft assemblies resolved into 100 or fewer contigs before further reduction to <72 contigs after scaffolding (Table 1). The quality of the draft assemblies was improved with Medaka’s polishing resulting in *BUSCO* scores that increased from 68.6 to 89.7 (a 30.8% increase), 67.2 to 85.5 (a 27.2% increase), and 68.8 to 85.9 (a 24.8% increase) for StAPkA1H1, sks047, and sks048, respectively, with *BUSCO* completeness scores for the clinical isolates reaching 95% (Table 1). The observed increase in the number of contigs from 23.57 to 23.63 Mb (0.22% increase) for sks047 and 24.49 to 24.56 Mb (0.32% increase) for sks048 was likely due to the addition of relatively shorter reads (Table 1).

**TABLE 1:** Overview of assembly and quality metrics of the *de novo* assembled draft assemblies.

Isolate	Coverage	*De novo* assembly length (Mb)					Contigs/scaffolds/chromosomes					BUSCO completeness score (%)				
		Raw	Medaka	Pilon	RagTag	Complete	Raw	Medaka	Pilon	RagTag	Complete	Raw	Medaka	Pilon	RagTag	Complete
PKNH^a^	—	—	—	—	—	24.36	—	—	—	—	15	—	—	—	—	97.6
PKA1H1^b^	—	—	—	—	—	24.27	—	—	—	—	14	—	—	—	—	94.4
StAPkA1H1	225X	24.15	24.14	N/A	24.39	24.39	73	111	N/A	71	15	68.6	89.7	—	89.7	89.5
sks047	71X	23.57	23.63	23.64	24.17	24.17	100	116	116	69	15	67.2	85.5	95.7	95.9	95.9
sks048	65X	24.49	24.56	24.57	24.81	24.81	74	94	94	50	15	68.8	85.9	95.7	95.7	95.6

Legend to Table 1: Quality improvements in the three *de novo* draft assemblies StPkA1H1, sks047, and sks048 were achieved by polishing with Medaka (Oxford Nanopore Technologies, 2019) and Pilon (Walker et al., 2014), checks for chimeric contig and scaffolding with RagTag (Alonge et al., 2019), and annotation of the draft assemblies with Companion (Steinbiss et al., 2016). The published P. knowlesi PKNH and PkA1H1 reference genomes generated from experimental lines were available in their complete forms. Information on raw reads and assembly was not available for comparison here.

a Pain et al.(2008).

b Diez Benavente et al.(2017).

The combination of previously sequenced Illumina reads data with 34x and 166x short read coverage for sks047 and sks048, respectively, offered the opportunity for Pilon polishing the newly generated ONT sequence data for the clinical isolates. Pilon polishing resulted in improved *BUSCO* scores with sks047 seeing an 11.9% improvement (85.5–95.7) and sks048 showing an 11.4% improvement (85.9–95.7) (Table 1). Although Pilon did not change the number of contigs, both sks047 and sks048 saw a total length increase of 0.05% and increases in *BUSCO* scores. Additional Illumina sequencing was not available for StAPkA1H1, and Pilon polishing was not possible.

Scaffolding, chromosome structuring, and subsequent annotation initially proved difficult due to large sections of chromosomes 2 and 3 consistently being incorrectly placed in chromosomes 14 and 13, respectively. These large-scale inconsistencies were the result of contig chimers and were minimized or entirely corrected by de-chimerization using RagTag. Chromosomes corrected by RagTag retained regions of variability for the draft assemblies, although RagTag did not provide a complete solution in resolving all variable sequences (Supplementary Figures S1, S2). In addition, it is possible that RagTag did not entirely retain highly variable regions such as telomeric regions that may have resulted in loss of coverage of genes positioned at extreme chromosomal boundaries (Supplementary Figure S1).

### Genome Annotation and Gene Content

Companion software resolved all three nuclear genomes, StAPkA1H1, sks047, and sks048, into 15 chromosomes–14 Pk chromosomes and 1 “bin” or “00” chromosome (chr 00) holding sequence fragments which could not be confidently placed by the Companion pipeline (Table 2). Each draft genome was assigned a similar or greater number of coding genes than the *P. knowlesi* PKNH reference genome (5327 genes) when full protein-coding genes and pseudogenes annotated with predicted function (implying missing “start” and/or “stop” codons) were combined. The StAPkA1H1 draft assembly had 5358 genes (4385 coding +973 pseudogenes), while the patient isolate draft genomes sks047 and sks048 had 5327 genes (4886 coding +441 pseudogenes) and 5398 genes (4904 coding +494 pseudogenes), respectively (Table 2). Non-coding genes were also found in all three draft genomes, including multiple small nuclear RNA (snRNA) (Supplementary File S1). *P. knowlesi schizont-infected cell agglutination variant antigen* (*SICAvar*) and the *Knowlesi-Interspersed Repeats* (*kir*) multiple gene families were annotated in each draft genome (Table 2). There were consistently fewer *kir* gene family members in the draft genomes derived from the clinical isolates sks047 and sks048 with 26 and 25 *kir* genes, respectively, compared with 51 *kir* genes in the experimental cultured line StAPkA1H1 and 56 in the published PKNH reference genome (Table 2). It is unlikely that this is a result of assembly error given that StAPkA1H1 and the clinical isolates sks047 and sks048 were sequenced and *de novo* assembled in parallel using the same methodologies with the exception of Pilon polishing for StAPkA1H1.

**TABLE 2:** Summary of the complete *de novo* draft genomes compared to the published *P. knowlesi* PKNH and PkA1H1 reference genomes.

Isolate	Complete assembly length (Mb)^a^	Contigs	Chromosomes	N50 (Mb)	N count	Gaps	Genes^b^	Total pseudo-genes	Shared orthologous clusters with reference	Unique orthologous clusters	Singleton clusters	KIRs	*SICAvars* ^c^		
													T1	T2	SDM’s
PKNH (Pain et al., 2008)	24.36	—	15	2.16	11,381	98	5327	12	—	—	—	56	89	20	127
PkA1-H.1 (Diez Benavente et al., 2017)	24.27	156	14	2.19	148,255	142	—	—	—	—	—	—	—	—	—
StAPkA1H1	24.39	71	15	2.13	288,598	127	5358	973	4172	3	62	51	191	15	88
sks047	24.17	69	15	2.09	544,896	109	5327	441	4666	9	82	26	115	9	181
sks048	24.81	50	15	2.21	283,076	84	5398	494	4664	11	100	25	153	7	196

Legend to Table 2: *SICAvar* domain fragments are found annotated across the genomes; combinations of these fragments can form complete *SICAvar* proteins, indicating the possibility of a larger number of *SICAvar* proteins present in native genomes. Gene data for reference PkA1H1 were unavailable.

a Total genome length excluding the mitochondrial and apicoplast genome sequences.

b Total number of coding genes and pseudogenes identified with a function.

c *SICAvar* type 1 (T1); *SICAvar* type 2 (T2); *SICAvar* single domain fragments (SDMs). Single domain fragments code for *SICAvar* protein fragments.

All three draft genomes had more *SICAvar* type 1 genes annotated than the reference PKNH genome. StAPkA1H1 had 191 *SICAvar* type 1 genes, sks047 had 115 *SICAvar* type 1 genes, and sks048 had 153 *SICAvar* type 1 genes. The reference PKNH genome is reported with 89 *SICAvar* type 1 genes (Table 2)*. SICAvar* gene fragments in each of the clinical isolate draft genomes, sks047 and sks048, outnumbered annotated *SICAvar* type 1 genes (Table 2). Conversely, the StAPkA1H1 draft genome had approximately half the number of *SICAvar* gene fragments compared with the clinical isolates and compared with StAPkA1H1 *SICAvar* type 1 genes (Table 2).

In regions of the draft genomes where gaps could not be resolved, contigs with evidence that they belonged together, either by long reads spanning them or by similarity to the reference, were scaffolded with N bases proportional to the gap size (Table 2). Higher N counts were observed in the three draft genomes generated here compared with the published reference genome (PKNH). In addition, sequences placed in the draft genome chr 00 may reflect the higher N counts in chromosomes 1–14. The chr 00 of StAPkA1H1 clustered with the PKNH reference chr 00 (Supplementary Figures S1,i) suggesting the StAPkA1H1 draft genome had a similar structure to the PKNH reference genome, including “unplaced” genes. In contrast, sks047 and sks048 chr 00 sequences were distributed across the reference genome, suggesting no single chromosome was more challenging to scaffold after de-chimerization (Supplementary Figure S2ii,iii). The number of gaps in the three draft genomes was variable but within the range of the PKNH reference genome (Table 2).

Orthologous genes were determined using a similarity approach by OrthoMCL in Companion and showed that all three draft genomes shared >4000 orthologs with the PKNH reference genome (Table 2). These orthologous genes can be considered as the core *P. knowlesi* gene set and are indicative of reliable and accurate assemblies (Table 2). In particular, draft genomes from the contemporary patient isolates sks047 and sks048 had >4600 shared orthologues with the PKNH reference genome (Table 2).

### Chromosome Structure

Dotplots of alignment of the three draft genomes show that they are syntenic with the PKNH reference regardless of gaps present in the genomes generated from patient isolates (Supplementary Figure S3). The unplaced sequences in chr00 account for at least 40% of gaps in the three draft genomes (Table 2). Indeed, each draft genome’s chromosome structure conforms to that of the PKNH reference genome with uniform coverage across the chromosomes in regions with no gaps (Supplementary Figure S4). This is also apparent in fragmented chromosomes, which retained the same chromosomal structure as PKNH (Supplementary Figure S5). While coverage remained largely uniform, structural variations (>10 kb), for example, duplications and inversions, were present in the draft assemblies as seen in duplications present in multiple chromosomes in sks047 and sks048 (Supplementary Figure S4b).

**FIGURE 2:**
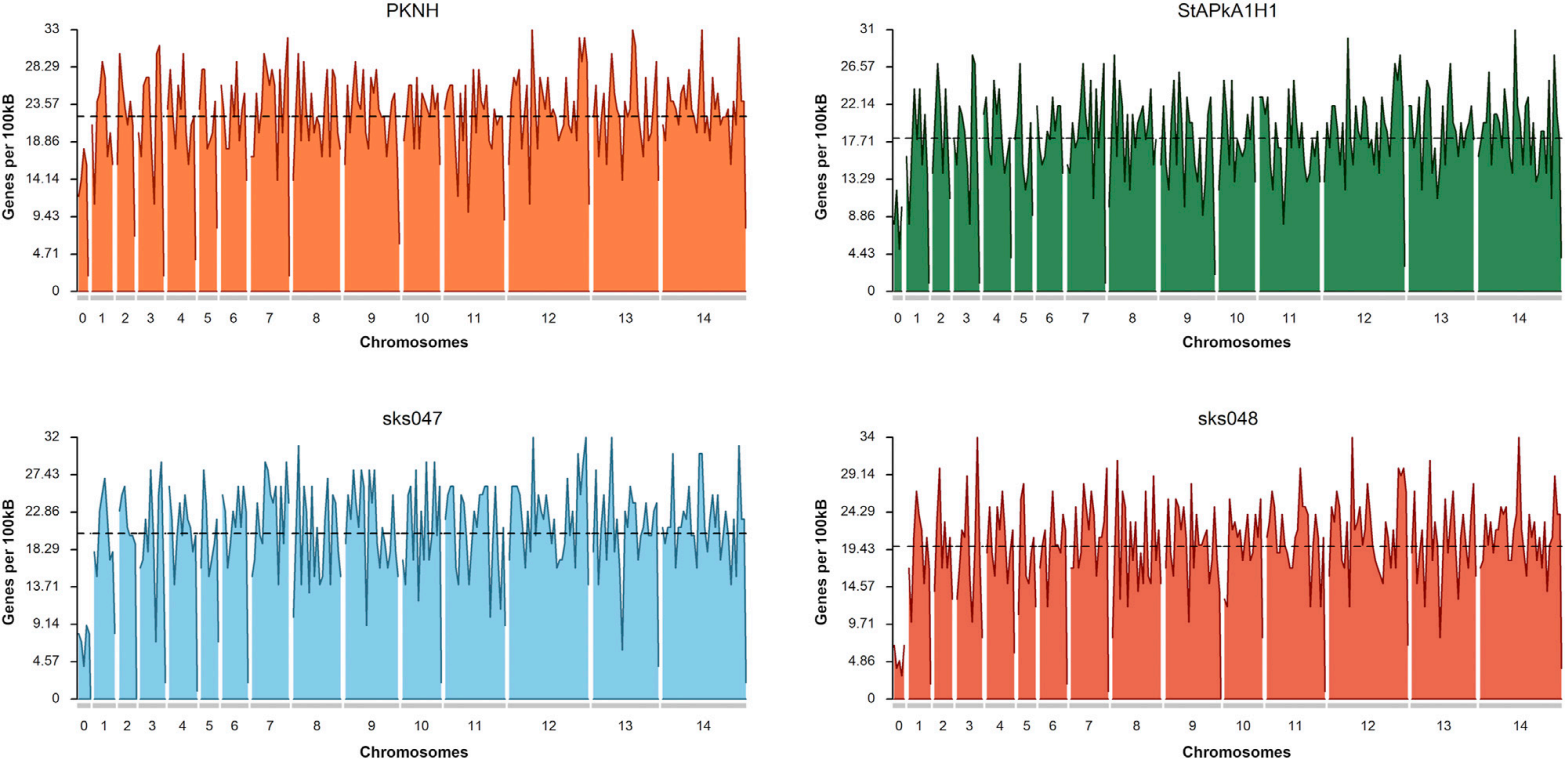
Gene density plots for the *P. knowlesi* PKNH reference genome, StPkA1H1, sks047, and sks048 draft genomes. Gene density is calculated based on the number of identified genes within a sliding window of 100 kb. Mean density shows the PKNH reference genome to have 22.05 genes per 100 kb, StAPkA1H1 to have 18.15, sks047 to have 20.25, and sks048 to have 19.8. Plots were generated using karyoploteR (Gel and Serra, 2017).

Additionally, inversions were present in almost every chromosome, often as inverted duplicate sequences, with the most striking instance observed in chromosome 5 of sks048 (Supplementary Figure S4a,iii), where multiple duplicated inversions were observed. Frameshifts were present across chromosomes in all of the draft genomes (Supplementary Figure S4b). Given the robust clinical isolate draft genome assembly, the frameshifts observed deserve further investigation. Associated gaps do not appear to have impacted the distribution of genes within the draft genomes (Figure 2). The mean annotated gene density shows the PKNH reference genome to have 22.05 genes per 100 kbp, StAPkA1H1 to have 18.15, sks047 to have 20.25, and sks048 to have 19.80 (Figure 2). Increased gene density may be achieved with manual pseudogene curation since mean gene density is inversely correlated with the number of pseudogenes, *p* = 0.003186 (Table 2).

With the exception of *SICAvar* and the *Interspersed Repeat (IR)* genes, analysis of the other multigene families reveals similar retention copy numbers in the three draft genomes and the PKNH reference (Table 3). Given the high similarity between the experimental lines StAPkA1H1 and PKNH in dotplots and other analyses, the total number of IR genes in the two different laboratory passaged lines, PKNH with 70 and StAPkA1H1 with 67, compared with clinical isolates, sks047 with 53 and sks047 with 52, may reflect gene retention through passive artificial passage. The clinical samples had fewer annotated *kir* genes than the experimental lines and in contrast have interspersed genes annotated as *P. vivax vir* that are absent in experimental lines (Table 3). Clinical isolates are effectively wild-type *P. knowlesi*, and the lower *kir* gene copy number and the presence of *vir*-like genes possibly reflect continual recombination and selection pressure during mosquito transmission in nature.

**TABLE 3:** Number of annotated protein copies of the multigene families identified.

Genes	Abbreviation	PKNH	StAPkA1H1	sks047	sks048
Circumsporozoite protein	*CSP/CS-TRAP*	2	2	2	2
Cytoadherence linked asexual protein/gene	*CLAG*	2	2	2	2
Duffy binding/Duffy-antigen protein [erythrocyte binding protein (alpha/beta/gamma)]	*DBP/DaBP* [*ERYBP(a/b/g)*]	3	3	3	3
Early transcribed membrane protein	*ETRAMP*	9	9	9	9
Knob-associated histidine-rich protein	*KAHRP*	1	1	1	1
*Knowlesi* interspersed repeats	*KIR*	56	51	26	25
*Knowlesi* interspersed repeats-like proteins	*KIRLP*	9	9	6	5
Vivax interspersed repeats	*VIR*	0	0	17	16
*Plasmodium* interspersed repeats	*PIR*	5	7	4	6
Merozoite surface protein	*MSP*	13	10	10	10
Multidrug resistance (-associated protein)	*MDRP/MDRaP*	4	3	3	3
Reticulocyte binding protein	*Pknbp/rbp*	2	2	2	2
Sporozoite invasion-associated protein	*SPIAP*	2	2	2	2
Tryptophan-rich antigen	*TrpRA*	29	29	30	29
ATP-binding cassette (ABC) transporter	*ABCtrp*	15	15	15	15
Apicomplexan apetala2 transcription factor	*ApiAP2*	29	28	28	28
Schizont-infected agglutination variant proteins	*SICAvar*	109	206	124	160

Chromosome positional analyses of the *kir* genes show varied distribution across chromosomes with only three *kir* genes represented in chr 00 in the clinical isolate draft genomes, perhaps supporting constrained *kir* gene copy number in nature (Supplementary Figure S6). *SICAvar* genes appear to be distributed across the genome, on all chromosomes, including the chromosomal extremities with more members annotated than previously reported by Pain et al. (2008), particularly on chromosomes 10, 11, and 12 (Supplementary Figure S7).

### Structural Variation

Following filtering for length, quality, and depth, reads-based structural variants (SVs) were called using the ONT SV pipeline and assembly-based SVs were called using Assemblytics (Nattestad and Schatz, 2016). The reads-based approach returned 1,316 and 1,398 SVs for sks047 and sks048, respectively (Table 4). The assembly-based approach returned 856 and 839 SVs for sks047 and sks048, respectively (Table 4). The reads-based approach is expected to return more variants due to a higher error rate in the raw reads used than in the collapsed assembly-based methodology.

**TABLE 4:** Summary of reads-based and assembly-based structural variants.

Isolate	Total SVs		Insertions		Deletions	
	Reads	Assembly	Reads	Assembly	Reads	Assembly
sks047	1,316	856	564	396	752	460
sks048	1,398	839	667	480	731	359

Legend to Table 4: Reads-based SV calling involved filtering draft genomes for quality, length, and depth before aligning sks047 and sks048 input reads against the StAPkA1H1 genome using the Oxford Nanopore structural variant pipeline. Assembly-based structural variants were called using Assemblytics (Nattestad and Schatz, 2016) by aligning the complete draft genomes of sks047 and sks048 against the StAPkA1H1 genome.

**FIGURE 3:**
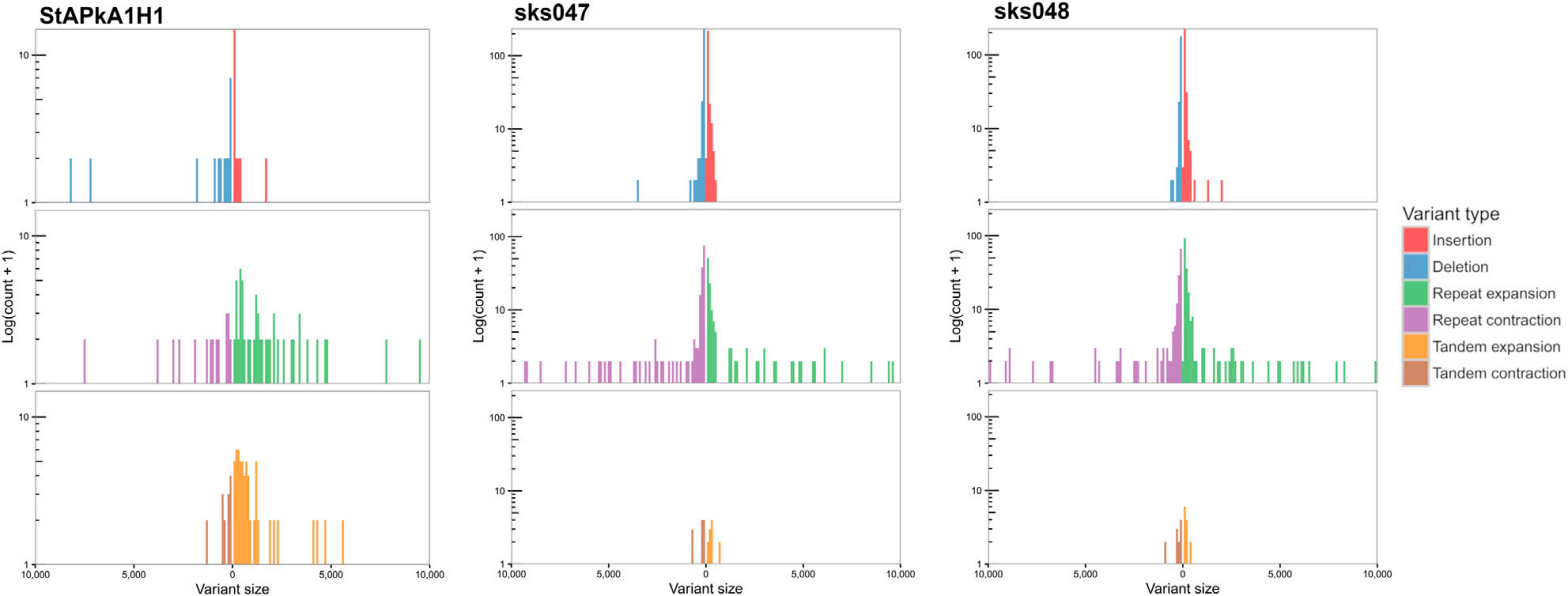
Assembly-based structural variation, size 50–10,000 bp, of StAPkA1H1, sks047, and sks048 draft genomes against the PKNH reference genome (Pain et al., 2008). Nucmer alignment was generated using parameters “—maxmatch−l 100−c 500” with default and Assemblytics parameters (Nattestad and Schatz, 2016). Expansions (green and orange) refer to insertions that occur within repeat or tandem variants, while contractions (purple and brown) refer to deletions in these regions. More variation is present in the tandem variants (brown and orange) of StAPkA1H1 than those of the draft clinical isolate genomes, sks047 and sks048. In comparison the clinical isolates show more variation in their repeat sequences with similar insertion and deletions (red and blue) and repeat expansion and contraction (green and purple) signatures.

SVs that exceeded the quality, length, and read depth threshold are distributed across the genome on all chromosomes within coding and non-coding regions. Within the 101 shared SVs, 68 were within annotated genes, including within the *SICAvar* and *kir* multigene family members (Supplementary Table S1). There were different variation signatures between the experimental cultured line StAPkA1H1 compared with the two clinical isolates, sks047 and sks048 (Figure 3). StAPkA1H1 had more tandem variants than the clinical isolates, sks047 and sks048. In comparison, the clinical isolates show more variation in their repeat sequences with similar insertion and deletion (red and blue) and repeat expansion and contraction (green and purple) signatures than StAPkA1H1 (Figure 3).

### Gene Duplication

**FIGURE 4:**
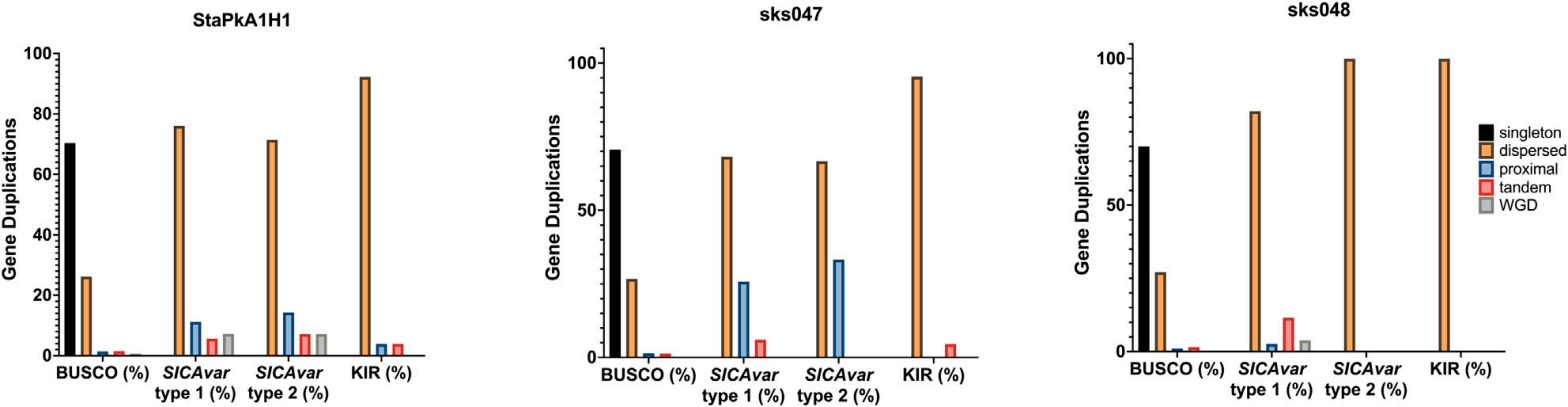
Gene duplication classes for the draft genome assemblies for StAPkA1H1 (experimental cultured line) and the clinical isolates sks047 and sks048. Gene duplication was quantified and classified using MCScanX (Wang et al., 2012) for all genes in each genome and identified as black bars, singleton (no identified duplication); orange bars, dispersed (>20 genes between the 2 candidate genes); blue bars, proximal (two identified duplicated genes with <20 genes between them); pink bars, tandem (duplication events next to each other); and green bars, segmental/whole genome duplication (WGD) (>4 co-linear genes with <25 genes between them). The gene pools for each genome were divided into *BUSCO* (core genome genes) for comparison with the genes making up the *SICAvar* type 1 or *SICAvar* type 2 or *kir* multiple gene families. The draft genomes, StAPkA1H1, sks047, and sks048, had roughly similar profiles for *BUSCO* genes. Singletons (dark blue bars) were absent from the multiple gene families for all of the draft genomes.

Gene duplication was quantified and classified using MCScanX (Wang et al., 2012). All genes within the draft genomes for the StAPkA1H1 cultured line and sks047 and sks048 clinical isolates were classified as either singleton (no identified duplication, proximal (two identified duplicated genes with <20 genes between them), dispersed (>20 genes between the 2 candidate genes), tandem (duplication events next to each other), and segmental/whole genome duplication (WGD) (>4 co-linear genes with <25 genes between them). To gain an insight into differences in gene duplication, duplication types were classified for the BUSCO eucaryotic core control gene population and for the PkSICAvar type 1, PkSICAvar type 2, and the kir multiple gene families in the three draft genomes, StAPkA1H1, sks047, and sks048 (Figure 4). The duplication profile of the control population BUSCO genes was well matched between each of the draft genomes and also to the BUSCO duplication profile for the PKNH reference genome (Mann–Whitney U test StAPkA1H1, *p* = 0.92; sks047, *p* = 0.67; sks048, *p* = 0.66; PKNH *p* = 0.40). Therefore, there were no observed excess duplication types for BUSCO genes (Figure 4). However, duplication profiles for the genes annotated *SICAvar* type 1, *SICAvar* type 2, and kir in the draft genomes, StAPkA1H1, sks047, and sks048, were markedly different from the BUSCO gene profiles with no evidence for singleton genes (Figure 4). When compared to 100 randomly obtained genes as a population, this result profile was statistically significant (Mann–Whitney U test, *p* < 1.0e−9).

### Positive Selection: Nonsynonymous (dN)/Synonymous (dS) Substitutions

In order to determine if the *SICAvar* type 1, *SICAvar* type 2, and *kir* genes are under selection pressure, the associated predicted proteins from each genome, StAPkA1H1, PKNH, sks047, and sks048, were translated into amino acid sequences and grouped into putative orthologous gene clusters containing *SICAvar* type 1 or *SICAvar* type 2 or *kir* or *BUSCO* (control group) using OrthoFinder. The amino acid sequences were aligned, and the alignments used to “backtranslate” into nucleotide coding sequences. The mean dN/dS values for *SICAvar* type 1, *SICAvar* type 2, *kir,* and *BUSCO* gene clusters were 2.40, 2.74, 2.35, and 0.35, respectively, and the differences were statistically significant (Wilcoxon rank sum test *p*-value adjustment method Bonferroni: *SICAvar* type 1, = 4.1e-08; *SICAvar* type 2 = 0.0063 and *kir*, *p* = 6.7e-13, Table 5 and Supplementary Figure S8).

**TABLE 5:** Non-synonymous versus synonymous (dN/dS) analysis of *SICAvar* type 1, *SICAvar* type 2, *kir*, and *BUSCO* gene clusters represented collectively in the StAPkA1H1, sks047, and sks048 draft genomes and the PKNH reference genome.

Cluster group	Cluster count (n)	Mean dN/dS per cluster	Standard deviation	Median	Inter quartile range
*BUSCO*	153	0.353	0.723	0.101	0.27
*SICAvar* type 1	15	2.4	1.31	2.37	1.86
*SICAvar* type 2	5	2.74	2.54	1.83	4.02
*kir*	26	2.35	1.19	1.99	1.5

Legend to Table 5: *SICAvar* type 1, *SICAvar* type 2, *kir* genes, and *BUSCO* (control groups) genes were translated into amino acid sequences and clustered into orthologous groups using OrthoFinder (Thorpe et al., 2018). The amino acid sequences were aligned and the alignments “backtranslated” into nucleotide coding sequences for subsequent dN/dS analysis using Codophyml (Gil et al., 2013). In order to avoid false-positive dN/dS results, the nucleic acid alignment was filtered to dis-allow gaps, insertions, and deletions, and the final filtered nucleotide alignments with three or more sequences per cluster, the minimum requirement for Codophyml (Gil et al., 2013), were subjected to dN/dS analysis. *SICAvar* type 1, *SICAvar* type 2, and *kir* genes had a statistically significantly greater dN/dS value when than *BUSCO* gene clusters (Wilcoxon rank sum test *p* value adjustment method Bonferroni: *SICAvar* type 1, *p* = 4.1e-08; *SICAvar* type 2, *p* = 0.0063; and *kir*, *p* = 6.7e-13).

### Genomic Organization of *SICAvar* Type 1, *SICAvar* Type 2 and *kir* Gene Family Members

**FIGURE 5:**
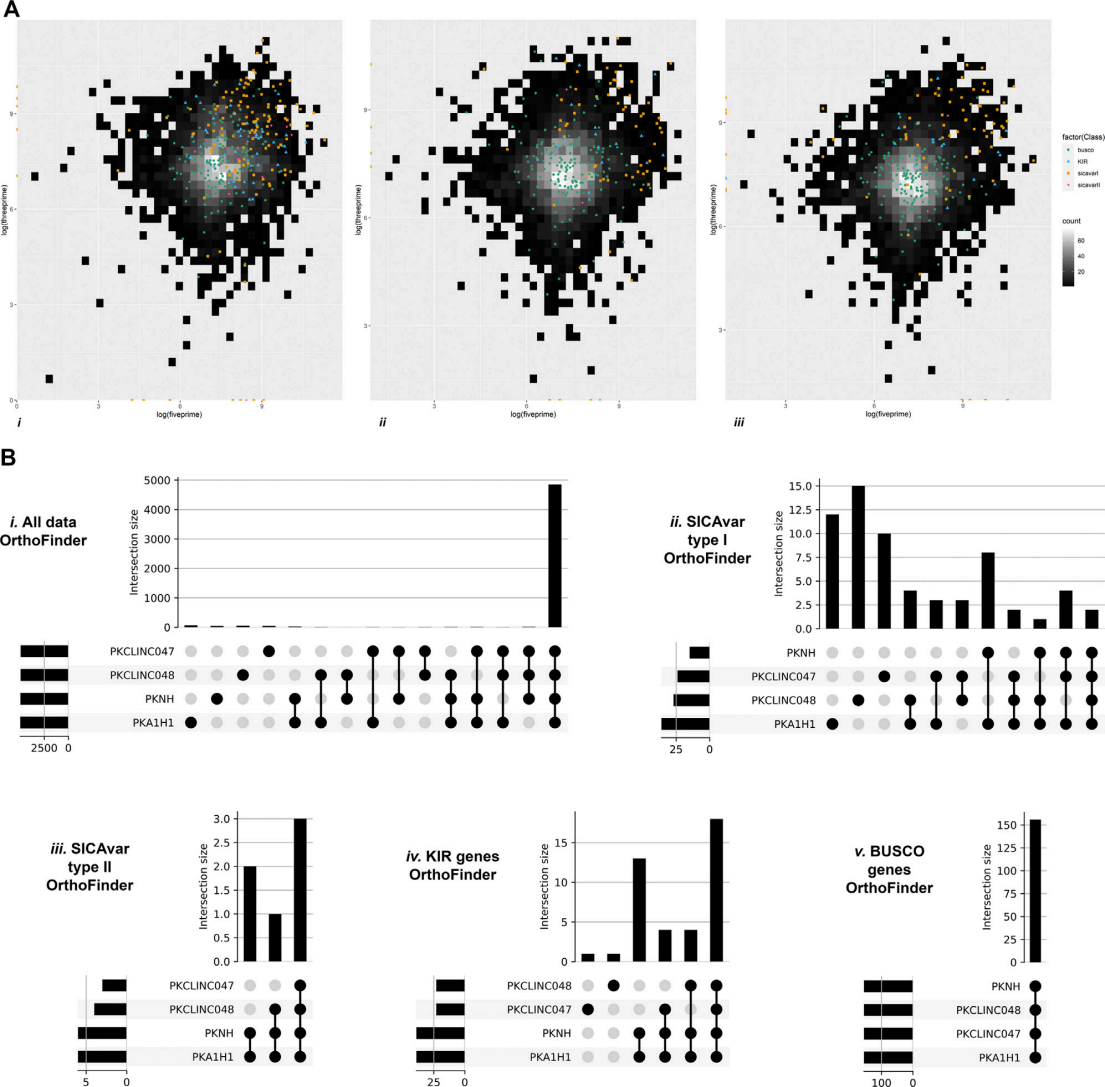
Genomic organization of multiple gene family members. Figure 5A Heatmap gene density plots showing 5’ against 3’ intergenic distances (log10) for the draft genomes. (i) StAPkA1H1 experimental cultured line, (ii) clinical isolate sk047, and (iii) clinical isolate sk048. Gene density for intergenic distances is represented by color scale ranging from black (low) to white (high, maximum of 60 genes per bin). Genes classed as *BUSCO* (green dots), *SICAvar* type 1 (orange squares), *SICAvar* type 2 (pink diamond), and *kir* (blue triangles) are shown. SICAvar type 1 and *kir* genes had a significantly greater distance to their neighboring genes than the *BUSCO* genes (Wilcoxon signed-rank test, Bonferroni *p*-value adjustment, *p =* < 1e-09), suggesting that these gene family members are in gene-sparse regions. Genes situated at the start or end of scaffolds were excluded from the analysis. Figure 5B OrthoFinder gene cluster outputs were visualized using “UpSets” to determine the membership of genes between clusters in the PKNH reference genome (Pain et al., 2008) and the draft genomes; StAPkA1H1 experimental cultured line, clinical isolate sk047, and clinical isolate sk048. All gene clusters (i), *SICAvar* type 1 gene clusters (ii), *SICAvar* type 2 gene clusters (iii), *kir* gene clusters (iv), and *BUSCO* gene clusters (v) are shown. The majority of all gene clusters were present in all isolates with the exception of *SICAvar* type 1 gene clusters with 10–15 *SICAvar* type 1 clusters being unique per isolate. For *kir* genes, the majority of clusters were shared between all isolates with the exception of a single unique *kir* gene cluster in each of sk047 and sk048. The majority of *SICAvar* type 2 genes were orthologues between all isolates with some not identified in sk047 and sk048.

*P. knowlesi SICAvar* type 1, *SICAvar* type 2, and the *kir* gene family members appear to be variable, rapidly evolving genes, yet they are distributed across chromosomes, potentially destabilizing the core genome. To investigate this further, the distance from one gene to its neighbor was quantified in both a 3 prime (3’) and 5 prime (5’) direction, excluding genes at the start or end of a scaffold. The values were subjected to further analysis using the *BUSCO* core genes for comparison (Figure 5A). With the exception of *SICAvar* type 2 in the 3’ direction, all of the *SICAvar* type 1, *SICAvar* type 2, and *kir* genes had a statistically significantly greater distance to their neighboring genes in both the 3’ and 5’ directions than *BUSCO* genes. In the 3’ direction, Kruskal–Wallis chi-squared = 272.15, df = 4, *p*-value < 2.2e-16. The Wilcoxon signed-rank test with Bonferroni *p*-value adjustment was *SICAvar* type 1 p = 2e-16, *SICAvar* type 2 *p* = 0.457, and *kir p* = 1.1e-10. In the 5’ direction, all distances for *SICAvar* and *kir* genes were significantly different to the *BUSCO* control population. Kruskal–Wallis chi-squared = 269.33, df = 4, *p*-value < 2.2e-16 with Wilcoxon signed-rank test, Bonferroni *p*-value adjustment in comparison to *BUSCO*: *SICAvar* type 1 *p* = 2e-16, *SICAvar* type 2 *p* = 0.00123, and *kir p* = 3.6e-09. The distribution of potentially destabilizing highly evolving *SICAvar* and *kir* genes across chromosomes in gene-sparse regions of the *P. knowlesi* genome would offer protection to core genes.

OrthoFinder gene cluster outputs were further visualized using “UpSets” to determine the membership of genes within each cluster. The majority of all gene clusters were present in all isolates with the exception of *SICAvar* type 1 clusters with between 10–15 unique *SICAvar* type 1 clusters per isolate (Figure 5B). For *kir* genes, the majority of clusters were shared between all isolates with the exception of a single unique *kir* gene cluster in each of sk047 and sk048. The majority of *SICAvar* type 2 genes were orthologues between all isolates with some not identified in sk047 and sk048 (Figure 5B).

## Discussion

Here, we demonstrate the utility of accessible, portable, and affordable PCR-free long-read ONT MinION sequencing to *de novo* assemble *P. knowlesi* genomes from small clinical samples, essentially wild-type parasites. The new genome sequences are robust and add context to our understanding of *P. knowlesi* genome structure, organization, and variability.

Three *Plasmodium knowlesi* draft genomes were assembled from two *P. knowlesi* clinical isolates (sks047 and sks048), and the other was a control genome from the *P. knowlesi* A1-H.1 (StAPkA1H1) experimental cultured line (Moon et al., 2013). Comparison of the *de novo* StAPkA1H1 genome assembled here with the *P. knowlesi* A.1-H1 genome generated using Illumina and PacBio platforms (Benavente et al., 2018) and the *P. knowlesi* reference genome PKNH (Pain et al., 2008) demonstrated that our sequencing platform and subsequent assembly pipeline produced robust and reliable *de novo P. knowlesi* genome sequences.

The two clinical isolates (sks047 and sks048) and the control (StAPkA1H1) resolved into 14 chromosomes as expected for *Plasmodium* spp. and one “bin” chr00. The PKNH reference genome also resolves into 14 chromosomes and one chr00 where 1.73% of the total sequence comprising 62 genes was assigned (Pain et al., 2008). Chr00 of StAPkA1H1, sks047, and sks048 contain 1.59%, 2.09%, and 1.94% total sequence length with 18, 35, and 25 genes, respectively. Failure of sequences to pass quality thresholds would be expected to be randomly distributed genome-wide as observed in sks047 and sks048 chr00 sequences. The observed clustering of StAPkA1H1 chr00 with PKNH chr00 is difficult to explain unless *de novo* chromosome structuring was being overridden, forcing StAPkA1H1 contigs into a chr00 to fit the pattern set by the PKNH reference genome (Assefa et al., 2015; Steinbiss et al., 2016).

During chromosome structuring, we found that the minimap2 alignment function of RagTag was unable to resolve chimeric contigs for sks047, sks048, and StAPkA1H1, perhaps as a function of the algorithm heuristics in minimap2 or localized flaws in our pipeline. Consequently, sections of sks047 chromosomes 02 and 03, which were incorrectly placed in chromosomes 14 and 13 due to chimeric contigs, were successfully corrected using the nucmer aligner function of RagTag.

In general, RagTag struggled to resolve regions of low complexity and high variability, such as telomeric regions, although we report predicted genes within these telomeric regions, including some members of the *SICAvar* gene family and those described by Lapp et al. (2018). More strikingly, the *Duffy-binding protein* and *TrpRA* genes are almost exclusively located at the extreme ends of the *de novo* assembled genomes presented here. Indeed, Otto et al., 2018 reported that Companion, as used here, can construct *Plasmodium* chromosomes in their entirety (Otto et al., 2018).

The published PKNH *P. knowlesi* genome (Pain et al., 2008) and *de novo* assembled StPkA1H1 genome have a similar compliment of Interspersed Repeat (IR) genes while the clinical samples are similar to each other but quite different to the experimental parasite lines. The clinical isolates have approximately half the number of *kir* genes compared with PKNH and StPkA1H1. Unexpectedly, the clinical isolates have IR genes annotated as *P. vivax* (*vir*) that are absent in PKNH and StPkA1H1. The most parsimonious explanation for this difference is that the data from *P. vivax vir* genes are derived from clinical samples, and there are no well-established experimental lines for *P. vivax*. Published *virs* may more closely reflect IR diversity accrued in contemporary parasites from the Old World monkey parasite clade that includes *P. knowlesi* and *P. vivax* (Singh et al., 2004). Nonetheless, it is an interesting observation that deserves further investigation.

*BUSCO* genes, with a similar duplication composition in the sks047, sks048, and StAPkA1H1 draft genomes, were used to compare duplication profiles for *SICAvar* type 1, *SICAvar* type 2, and *kir* gene family members that code for antigenically variable parasite proteins expressed on the surface of infected host red blood cells. The *SICAvar* type 1*, SICAvar* type 2, and *kir* gene population in all three draft genomes had significantly different duplication profiles when compared with 100 randomly selected genes (Mann–Whitney U test: *p* << 0.001). This suggests that the parasite genome tolerates high levels of duplication at these loci. Non-synonymous substitution over synonymous substitution (dN/dS) values greater than 1.0 are associated with positive selection pressure. *BUSCO* core eukaryotic genes are not thought to be under undue selection pressure and were used as a control gene set in dN/dS analysis to investigate selection pressure on clusters containing *SICAvar* type 1, *SICAvar* type 2, and *kir* genes. The mean dN/dS was 2.4 for *SICAvar* type 1 gene clusters, 2.74 for *SICAvar* type 2 clusters, and 2.35 for *kir* gene clusters while dN/dS scores for *BUSCO* gene clusters was 0.35, suggesting that the *SICAvar* type 1*, SICAvar* type 2, and *kir* gene populations are under strong positive selection pressure. Given that the protein products of these multiple gene family members are expressed at the forefront of parasite–host interactions, positive selection in addition to the gene duplication profiles observed would be expected to accommodate antigenic variability and increase the chance of parasite survival in a hostile host environment.

On the backdrop of signatures of change and variability observed and the potential for genome destabilization at these rapidly evolving loci, the distribution of the *SICAvar* and *kir* genes within chromosomes seemed counterintuitive. Indeed, the ability of *P. falciparum* to tolerate the highly evolving *PfEMP* 1 gene family members is explained by their positioning in the extreme sub-telomeric regions of chromosomes that support higher rates of recombination in comparison to relatively more conserved centromeric regions (Otto et al., 2018). In the case of *P. knowlesi*, we found the rapidly evolving *SICAvar* and *kir* genes positioned in otherwise gene-sparse regions of chromosomes. With the exception of *SICAvar* type 2 genes in the 3’ direction, *SICAvar* and *kir* genes had significantly greater distances to neighboring genes in the 3’ and 5’ directions than *BUSCO* genes. Gene-sparse regions tolerate transposon and repetitive rich regions necessary to generate antigenic variability at these important loci while reducing the probability of impacting essential core gene function. Similar protective positioning of highly evolving genes is found in plant pathogens, for example, nematodes (Eves-van den Akker et al., 2016), aphids (Thorpe et al., 2018), phytophthora (Haas et al., 2009; Thorpe et al., 2021), and fungi (Dong et al., 2015). The capacity of some genomic regions to generate more variation than others is poorly understood, but in the field of plant pathogens, it is termed “the two speed genome” (Dong et al., 2015). The “two speed genome” concept may well describe accumulation of multiple gene family members in *Plasmodium* species, particularly the *var* genes, and consequently provide a biological model with which to explain antigenic variation in *P. knowlesi*.

To further demonstrate *SICAvar* type 1 genetic divergence, UpSet visualization of each of the draft genomes assembled here had between 10 and 15 unique *SICAvar* type 1 gene clusters, more than any other orthologous gene cluster. Indeed, only two *SICAvar* type 1 geneclusters were shared among the draft genomes. In contrast, the *kir* genes were less divergent with only one unique gene-cluster in sk048 and in sk047 with most *kir* gene clusters common between clinical isolates and experimental lines.

The ability to generate variation and maintain fitness is fundamental to pathogen–host interactions. The pathogen needs a lifespan long enough to replicate, disseminate, and maintain germlines. The ability to generate diversity on genes that code for “exposed” proteins while protecting core gene function increases the chance of pathogen survival. The strong signatures of positive selection pressure and gene duplication on the *P. knowlesi SICAvar* type 1, *SICAvar* type 2, and *kir* genes irrefutably demonstrate their importance in the fitness and evolution of this particular pathogen. The methods developed here will be used to generate *P. knowlesi* genomes from patient isolates with matched metadata for parasite genome-wide disease association analyses. Experimental *Plasmodium knowlesi* is particularly receptive to genome editing, facilitating allele-specific phenotyping (Mohring et al., 2020). Parasites edited with clinically relevant disease-associated alleles can be taken forward and characterized *in vitro* and *in vivo* for cause and effect. In essence, *P. knowlesi* as the agent of zoonotic malaria and as an experimental parasite has the potential to closely model severe malaria pathophysiology.

On a broader landscape, an opportunity is presented to the global research community to generate genome-wide data from clinical infections to add “real world” context to malaria research.

## Data Availability

The datasets presented in this study can be found in online repositories. The names of the repository/repositories and accession number(s) can be found below: Sequencing data can be found at NCBI SRA BioProject, accession no: PRJNA799698; Scripts used to generate the data in this project are available in github: https://github.com/damioresegun/Pknowlesi_denovo_genome_assembly and https://github.com/peterthorpe5/plasmidium_genomes.
