# Supplementary material for: De Novo Assembly of Plasmodium knowlesi Genomes From Clinical Samples Explains the Counterintuitive Intrachromosomal Organization of Variant SICAvar and kir Multiple Gene Family Members
Source: Front Genet. 2022 May 23;13:855052. doi: 10.3389/fgene.2022.855052 (PMC9169567; doi:10.3389/fgene.2022.855052)
Supplement: Supplementary file 1 [file DataSheet1.zip › Suppl Information/SI_Fig5-8.pdf]

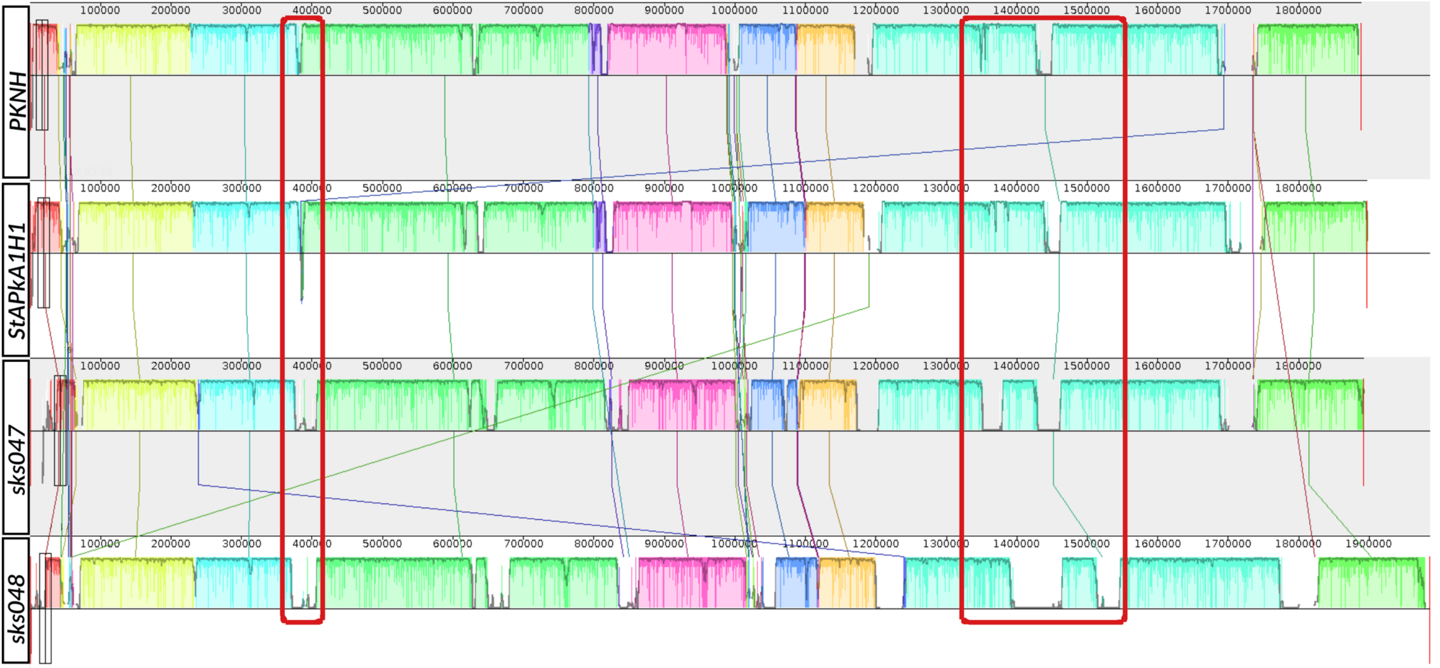

SI Fig 5. Mauve plot of chromosome 08 for StAPkA1H1, sks047, sks048 and the PKNH reference genome. Chromosome 8 of the PKNH reference shows more fragmentation than other chromosomes in the genome which may have influenced the chromosome structure inferred for the draft genomes generated here. Extensive mosaicism has been described in *P. knowlesi* chromosome 8 due to an overrepresentation of genes expressed in the mosquito stage of the parasite's life cycle. Regions of low coverage are still apparent in the draft genomes compared with the PKNH reference genome (red boxes).

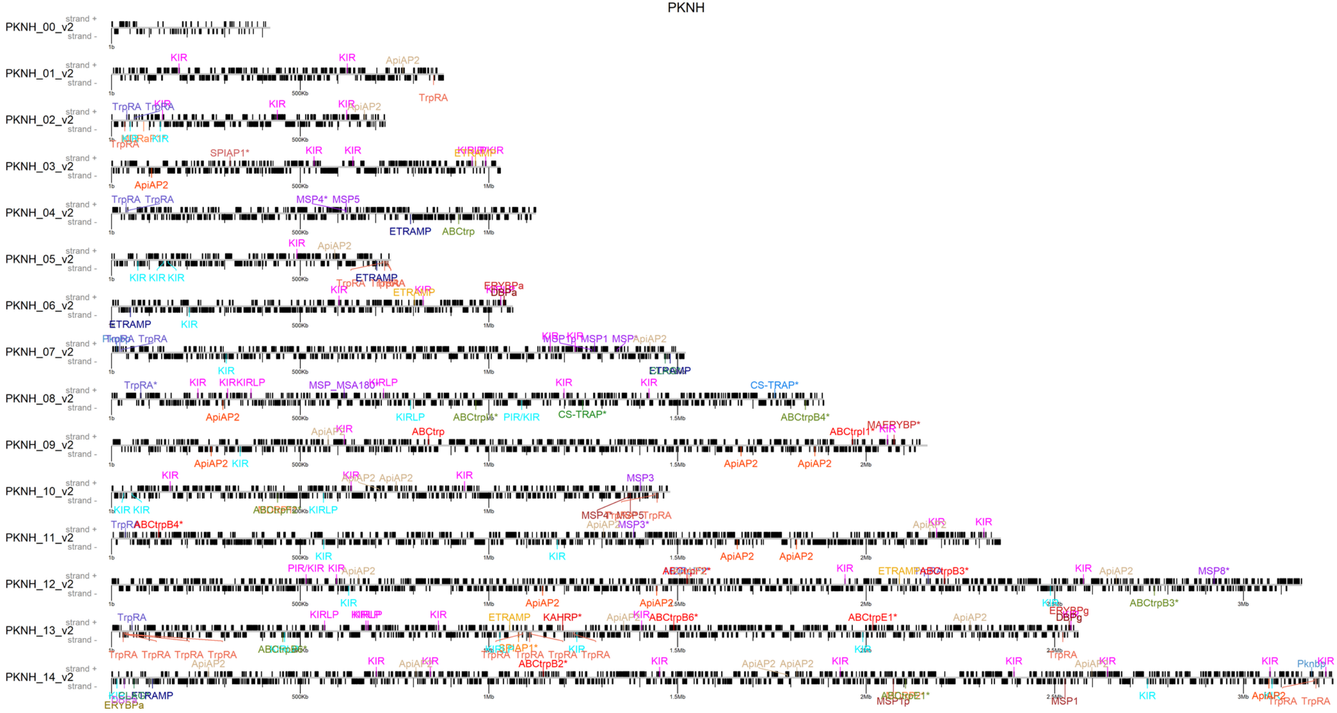

SI Fig 6A. *P. knowlesi* PKNH reference genome positioning of non-*SICAvar* multigene family members are shown using karyoploteR. Genes are shown as black squares marked along the chromosome linear map. Genes on the positive strand appear above the map line and those on the negative strand below. Identified members of select multigene families are given and colour coded based on being on the positive or negative strand e.g *TrpRA* on the positive strand is slate blue and coral on the negative strand. The *SICAvar* gene family members are presented separately in **SI Fig 7**.

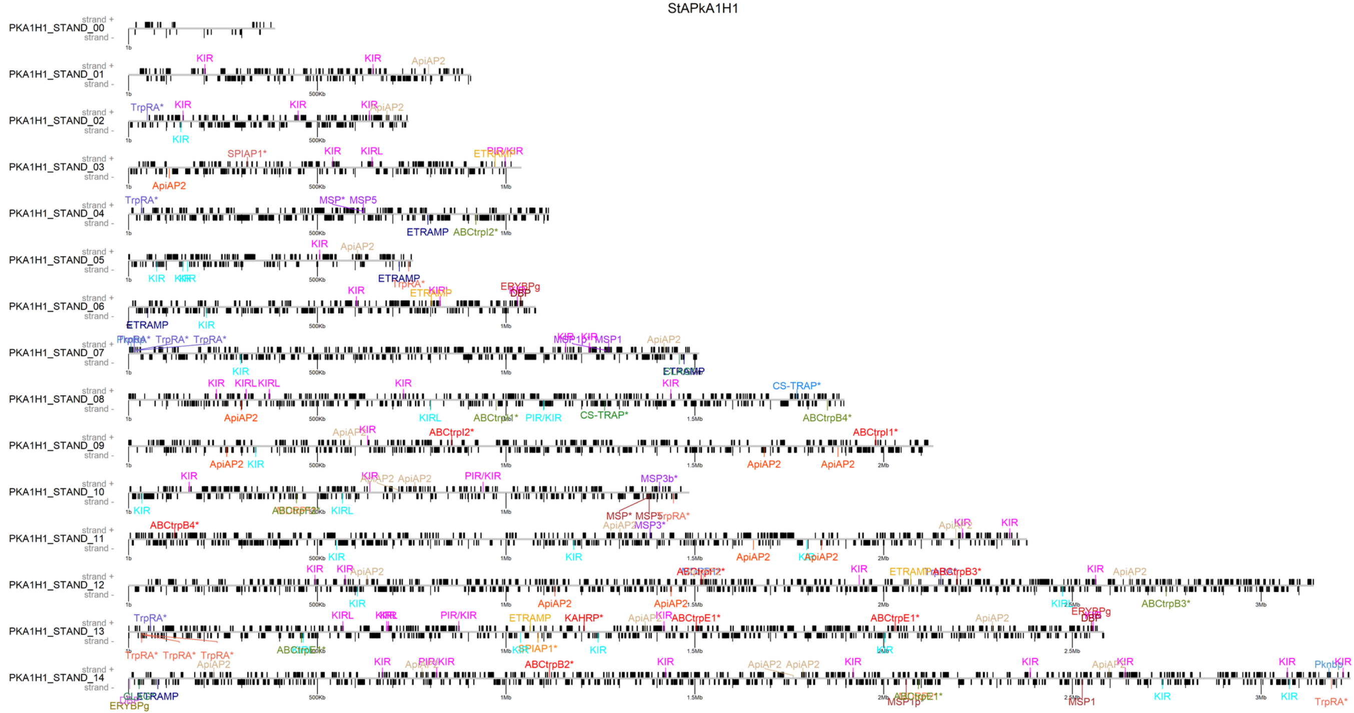

SI Fig 6B. StAPkA1H1 positioning of non-*SICAvar* multigene family members are shown using karyoploteR. Genes are shown as black squares marked along the chromosome linear map. Genes on the positive strand appear above the map line and those on the negative strand below. Identified members of select multigene families are given and colour coded based on being on the positive or negative strand e.g *TrpRA* on the positive strand is slate blue and coral on the negative strand. The *SICAvar* gene family members are presented separately in **SI Fig 7**.

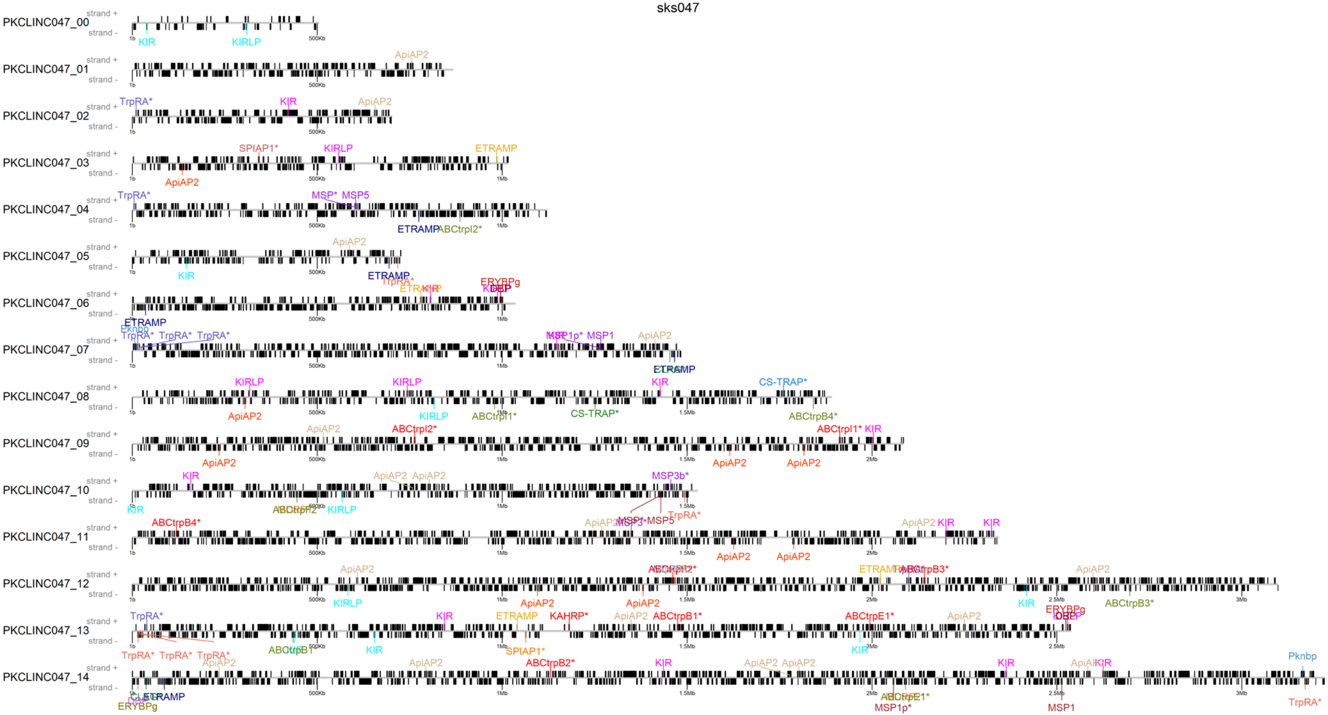

SI Fig 6C. sks047 positioning of non-*SICAvar* multigene family members are shown using karyoploteR. Genes are shown as black squares marked along the chromosome linear map. Genes on the positive strand appear above the map line and those on the negative strand below. Identified members of select multigene families are given and colour coded based on being on the positive or negative strand e.g *TrpRA* on the positive strand is slate blue and coral on the negative strand. The *SICAvar* gene family members are presented separately in **SI Fig 7.**

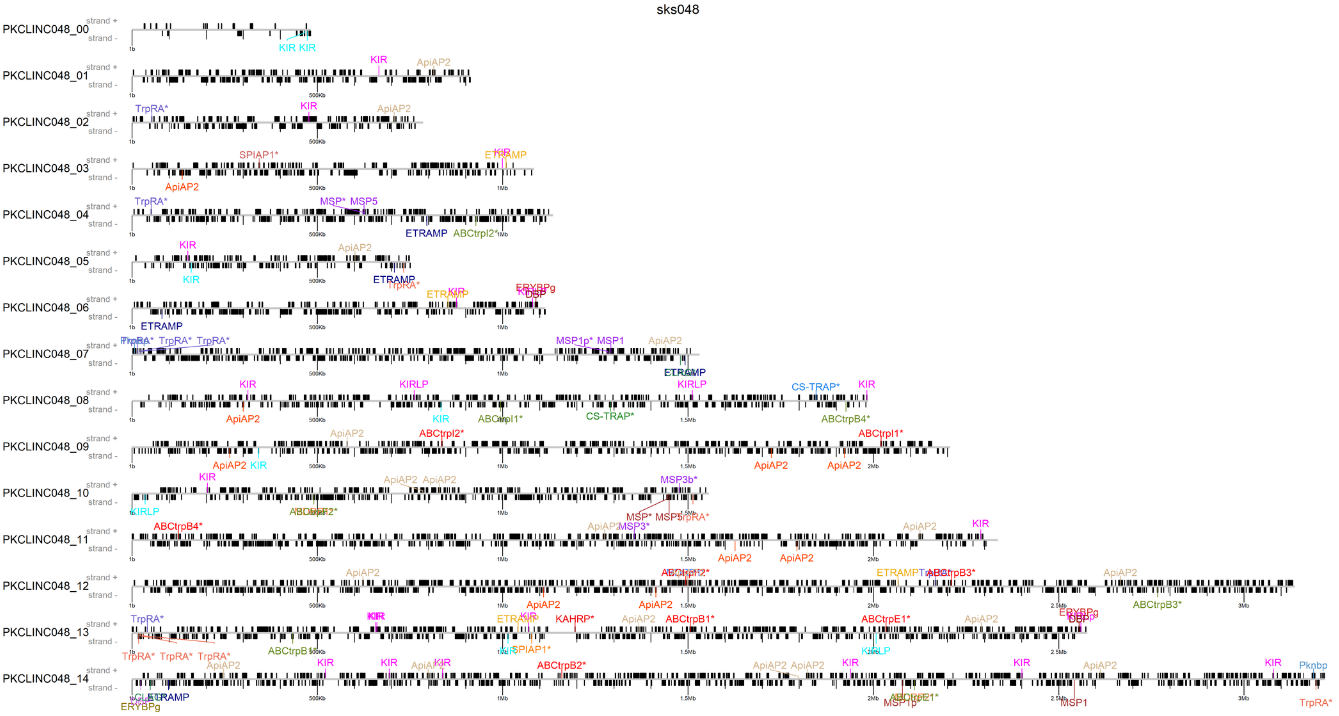

SI Fig 6D. sks048 positioning of non-*SICAvar* multigene family members are shown using karyoploteR. Genes are shown as black squares marked along the chromosome linear map. Genes on the positive strand appear above the map line and those on the negative strand below. Identified members of select multigene families are given and colour coded based on being on the positive or negative strand e.g *TrpRA* on the positive strand is slate blue and coral on the negative strand. The *SICAvar* gene family members are presented separately in **SI Fig 7.**

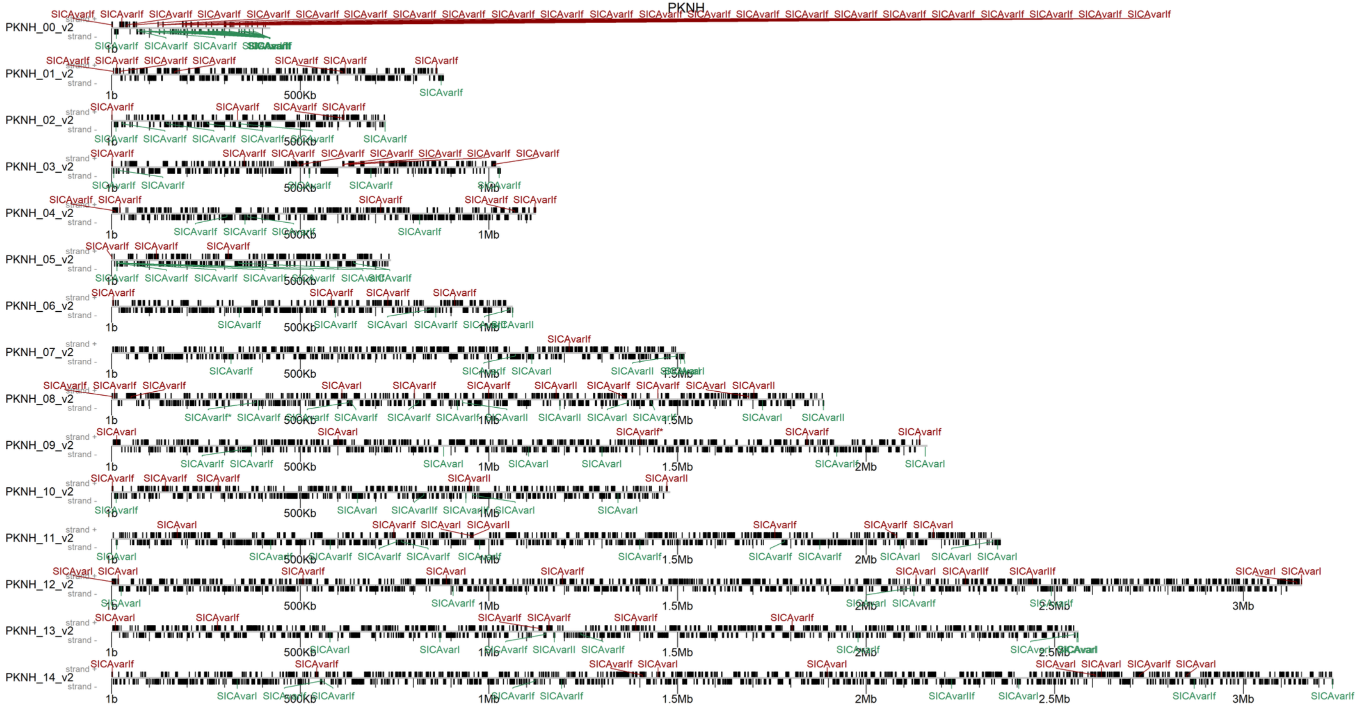

SI Fig 7A. *P. knowlesi* PKNH reference genome positioning of *SICAvar* multigene family members are shown using karyoploteR. Annotated genes are shown as black squares marked along the chromosome linear map. Genes on the positive strand appear above the map line and those on the negative strand below. *SICAvar* genes and gene fragments on the positive strand are in red font and on the negative strand in green font.

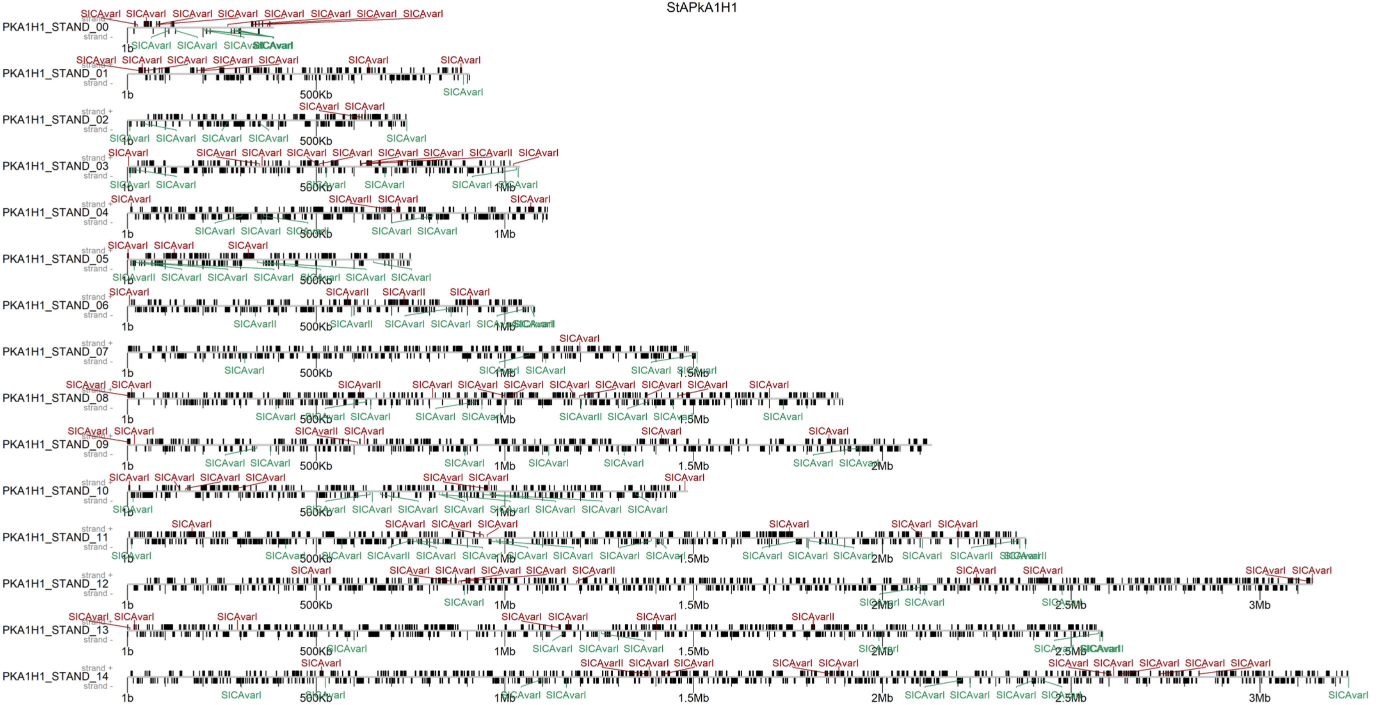

SI Fig 7B. StAPkA1H1 positioning of *SICAvar* multigene family members are shown using karyoploteR. Annotated genes are shown as black squares marked along the chromosome linear map. Genes on the positive strand appear above the map line and those on the negative strand below. *SICAvar* genes and gene fragments on the positive strand are in red font and on the negative strand in green font.

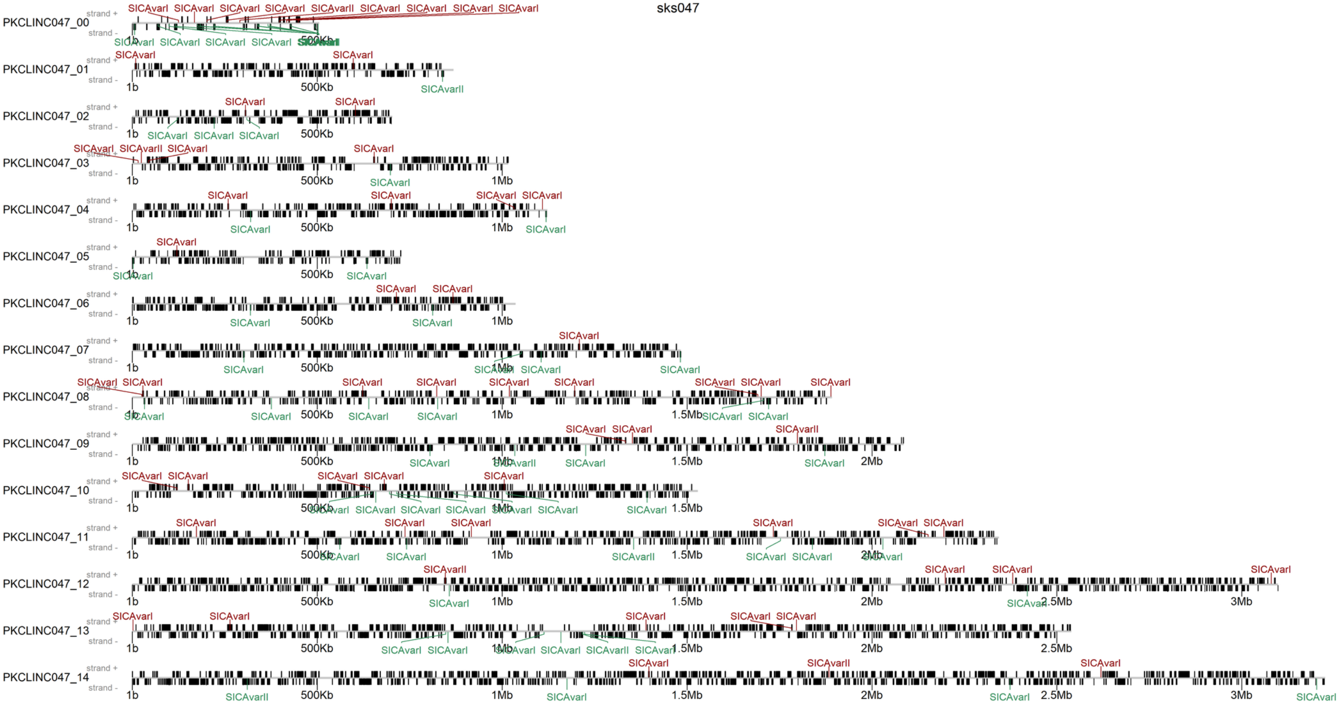

SI Fig 7C. sks047 positioning of *SICAvar* multigene family members are shown using karyoploteR. Annotated genes are shown as black squares marked along the chromosome linear map. Genes on the positive strand appear above the map line and those on the negative strand below. *SICAvar* genes and gene fragments on the positive strand are in red font and on the negative strand in green font.

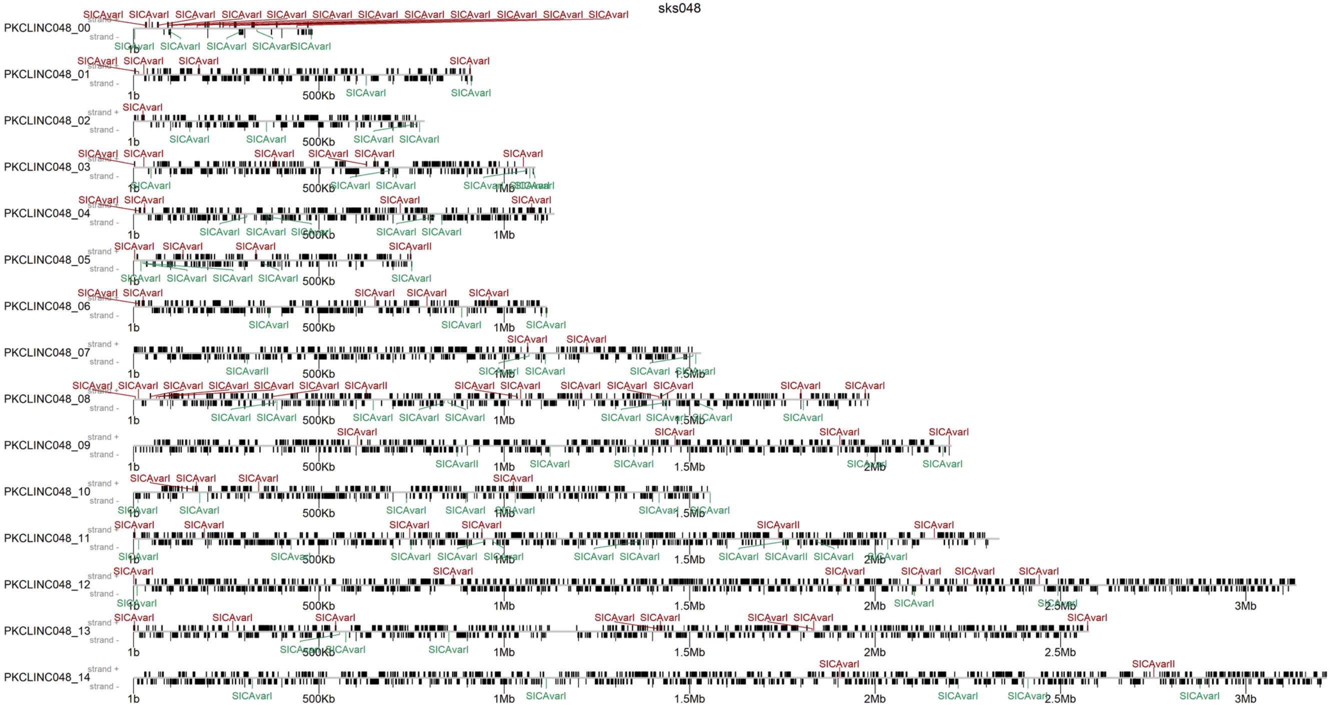

SI Fig 7D. sks048 positioning of *SICAvar* multigene family members are shown using karyoploteR. Annotated genes are shown as black squares marked along the chromosome linear map. Genes on the positive strand appear above the map line and those on the negative strand below. *SICAvar* genes and gene fragments on the positive strand are in red font and on the negative strand in green font.

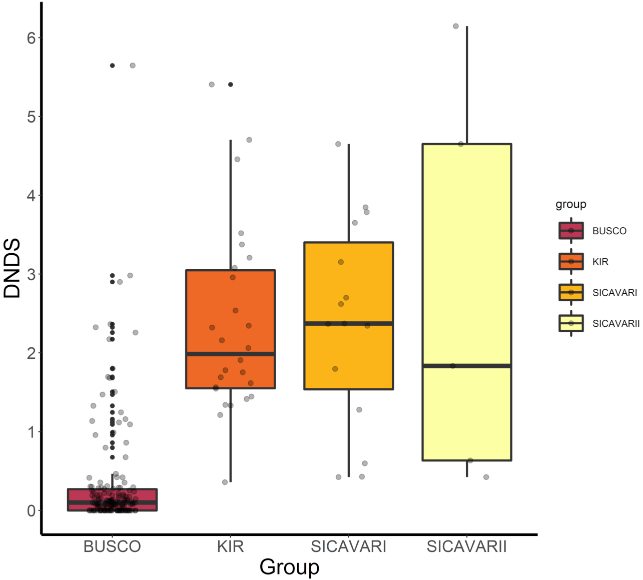

SI Fig 8. Box plot to represent dN/dS ratios for gene-clusters from each gene type: BUSCO; KIR; SICAvar type 1 and SICAvar type 2 in the combined dataset from draft genomes StAPkA1H1, sks047, sks048 and the PKNH reference. There were 154 BUSCO, 27 KIR, 15 SICAvar type 1 and 5 SICAvar type 2 gene clusters with mean number of genes 5, 8.59, 32.2 and 18.2 per cluster respectively. Clusters containing SICAvar type 1, or SICAvar type 2 or KIR genes had a statistically significant greater mean dN/dS value when compared to BUSCO gene clusters (Wilcoxon rank sum test *p*-value adjustment method Bonferroni: SICAvar type 1, = 4.1e-08; SICAvar type 2 = 0.0063 and KIR, *p* = 6.7e-13) suggesting these gene family members are under selection pressure.
