# Supplementary material for: De Novo Assembly of Plasmodium knowlesi Genomes From Clinical Samples Explains the Counterintuitive Intrachromosomal Organization of Variant SICAvar and kir Multiple Gene Family Members
Source: Front Genet. 2022 May 23;13:855052. doi: 10.3389/fgene.2022.855052 (PMC9169567; doi:10.3389/fgene.2022.855052)
Supplement: Supplementary file 1 [file DataSheet1.zip › Suppl Information/SI_Fig4b.pdf]

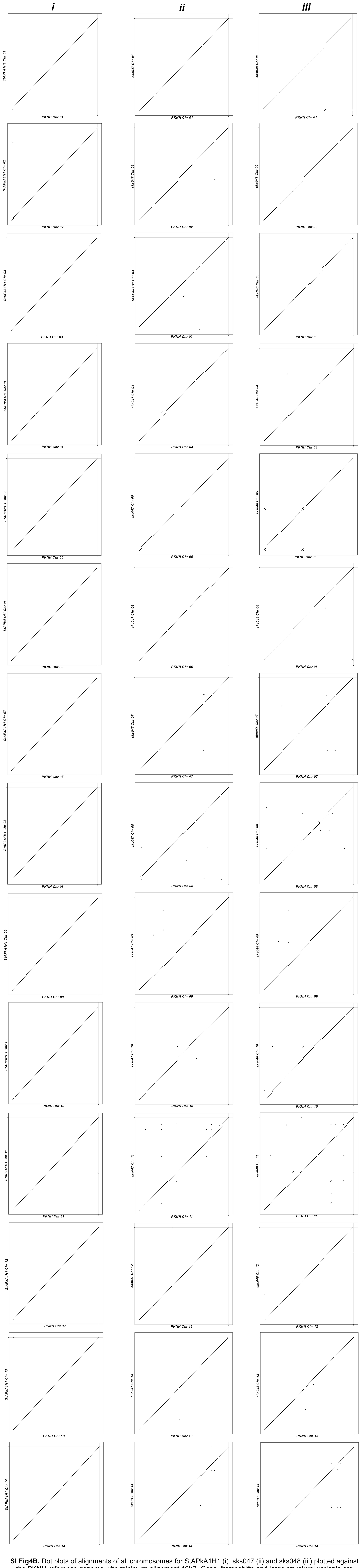

SI Fig4B. Dot plots of alignments of all chromosomes for StAPkA1H1 (i), sks047 (ii) and sks048 (iii) plotted against the PKNH reference genome with minimum alignment 10kB. Gaps, frameshifts and large structural variants are dispersed across the draft genomes are shown.
