# Supplementary material for: De Novo Assembly of Plasmodium knowlesi Genomes From Clinical Samples Explains the Counterintuitive Intrachromosomal Organization of Variant SICAvar and kir Multiple Gene Family Members
Source: Front Genet. 2022 May 23;13:855052. doi: 10.3389/fgene.2022.855052 (PMC9169567; doi:10.3389/fgene.2022.855052)
Supplement: Supplementary file 1 [file DataSheet1.zip › Suppl Information/Supplementary Tables .docx]

Supplementary Table 1 Length of non-nuclear DNA content present in the P. knowlesi PKNH [1] and PkA1H1 [2] reference in comparison to the three generated draft genomes.

| **Isolate** | **Apicoplast length (bp)** | **Mitocondrial length (bp)** |
| --- | --- | --- |
| **PKNH (Ref)** | 30638 | 5957 |
| **PKA1H1 (Ref)** | 34438 | 5957 |
| **StAPkA1H1** | 35485 | 6075 |
| **sks047** | Unresolved | 7132 |
| **sks048** | 34475 | 6136 |

Supplementary Table 2 Comparisons of variant call format (VCF) files of sks047 and sks048 against StAPkA1H1 draft genome

| **SV call Approach** | **# Shared structural variants** | **# Structural variants unique to sks047** | **# Structural variants unique to sks048** | **# Shared structural variants within annotated genes** |
| --- | --- | --- | --- | --- |
| **Assembly-based** | 101 | 755 | 738 | 68 |
| **Reads-based** | 2 | 1412 | 1544 | 1 |

Legend to Supplementary Table 2: Comparisons were achieved after analysis using the intersect (isec) function of bedtools. Assembly-based SV calling approach utilised Assemblytics [3] to call variants between the isolate draft genomes sks047, sks048 and the StAPkA1H1 draft genome. Reads-based SV calling approach used input reads of the isolate draft genomes against the StAPkA1H1draft genome to call variants with the Oxford Nanopore Structural Variation pipeline.

1. Pain A, Bohme U, Berry AE, Mungall K, Finn RD, Jackson AP, et al. The genome of the simian and human malaria parasite Plasmodium knowlesi. Nature. 2008;455(7214):799-803. Epub 2008/10/10. doi: 10.1038/nature07306. PubMed PMID: 18843368; PubMed Central PMCID: PMCPMC2656934.

2. Benavente ED, de Sessions PF, Moon RW, Grainger M, Holder AA, Blackman MJ, et al. A reference genome and methylome for the Plasmodium knowlesi A1-H.1 line. Int J Parasitol. 2018;48(3-4):191-6. Epub 2017/12/21. doi: 10.1016/j.ijpara.2017.09.008. PubMed PMID: 29258833.

3. Nattestad M, Schatz MC. Assemblytics: a web analytics tool for the detection of variants from an assembly. Bioinformatics. 2016;32(19):3021-3. doi: 10.1093/bioinformatics/btw369.
