# Supplementary material for: De Novo Assembly of Plasmodium knowlesi Genomes From Clinical Samples Explains the Counterintuitive Intrachromosomal Organization of Variant SICAvar and kir Multiple Gene Family Members
Source: Front Genet. 2022 May 23;13:855052. doi: 10.3389/fgene.2022.855052 (PMC9169567; doi:10.3389/fgene.2022.855052)
Supplement: Supplementary file 1 [file DataSheet1.zip › Suppl Information/SI_Fig1-4a.pdf]

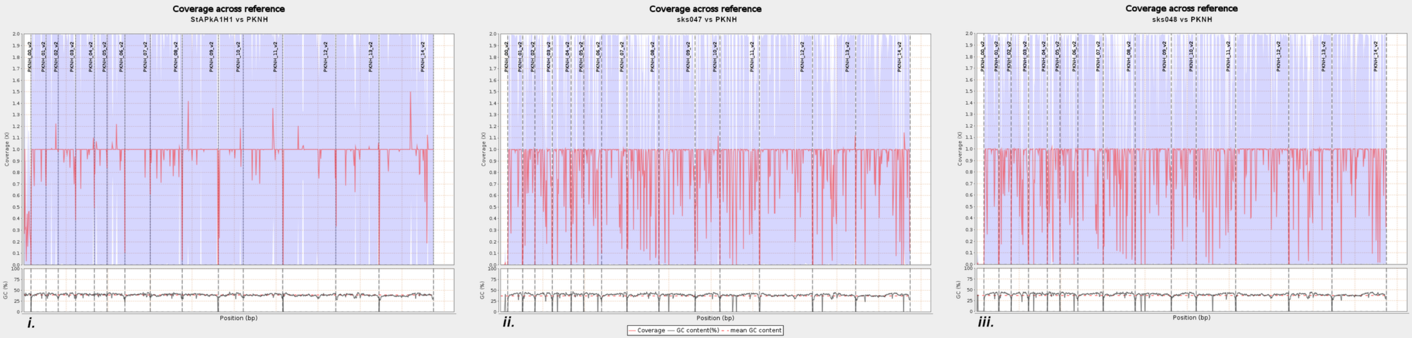

SI Fig 1. Whole genome coverage across chromosomes of the StAPkA1H1, sks047 and sks048 draft genomes against the PKNH reference genome. Coverage and plots generated using Qualimap are shown. The red trace shows troughs that indicate regions of low coverage. Coverage appears more stable in StAPkA1H1 (i) than in the clinical isolates sks047 (ii) and sks048 (iii) indicating higher variability in the contemporary P. knowlesi genomes than in the experimental line when compared with the reference

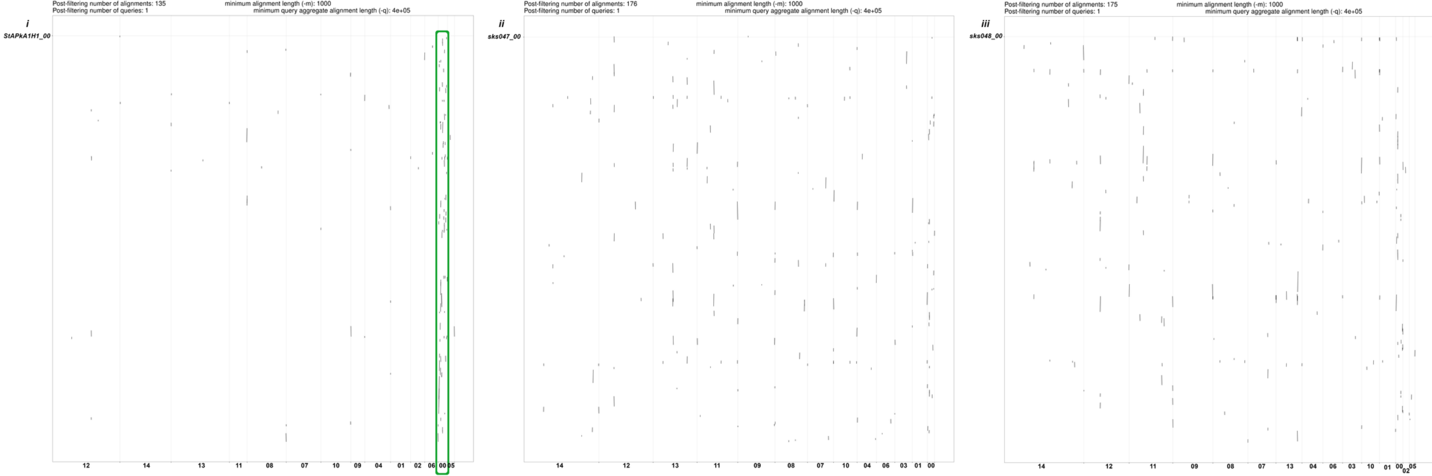

SI Fig 2. Alignments of chromosome 00 (bin) for StAPkA1H1, sks047 and sks048 against the whole PKNH reference genome. Minimap2 alignments of the bin chromosomes against the entire PKNH reference genome with a 1kbp alignment length filter. The ‘bin’ chromosomes contain sequence fragments that could not be confidently resolved into a particular chromosome during the scaffolding process. StAPkA1H1(i) shows a concentration of sequences aligned to the PKNH ‘bin’ chromosome 00 (green box), while no clustering is evident in sks047(ii) and sks048 (iii).

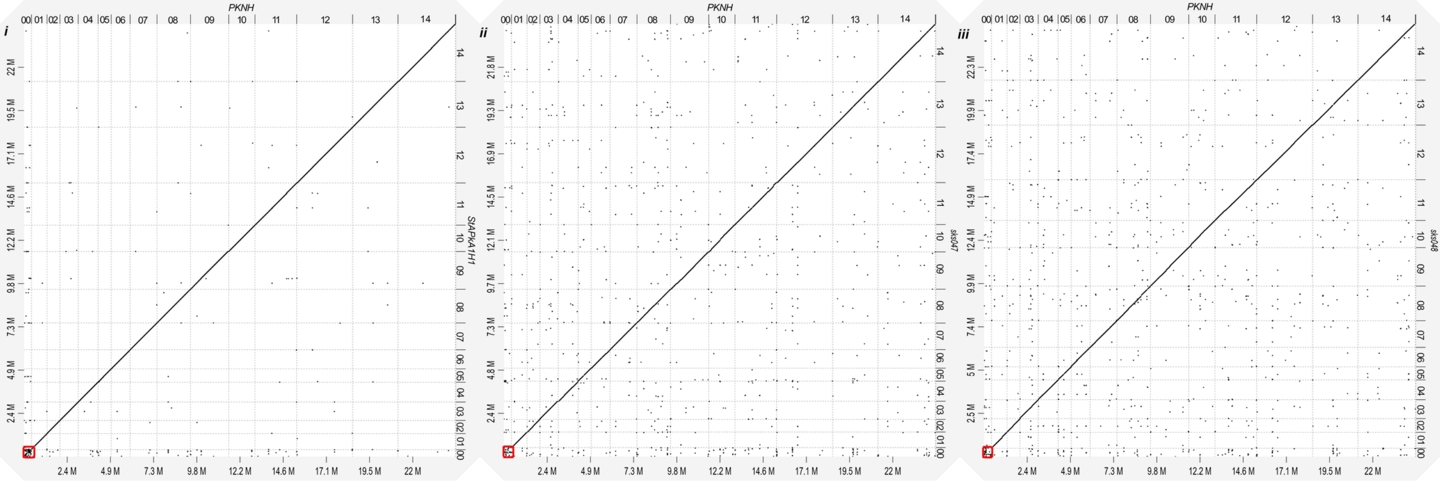

SI Fig 3. Whole-genome alignment of StAPkA1H1, sks047 and sks048 against the *P. knowlesi* PKNH reference genome. Dotplots to identify repetitions, breaks and inversions were generated from minimap2 whole draft genome alignments for StAPkA1H1(i), sks047 (ii) and sks048 (iii) using D-GENIES default settings. The PKNH chromosomes 00 – 14 are shown on the x-axes at the top and size given on the bottom in MB. Draft genome chromosomes 00 – 14 are shown on the right y-axes and size in MB on the left. The line indicates gene synteny between each draft and the PKNH reference genome. Red boxes show where the draft '00' chromosomes align with PKNH chromosome '00'.

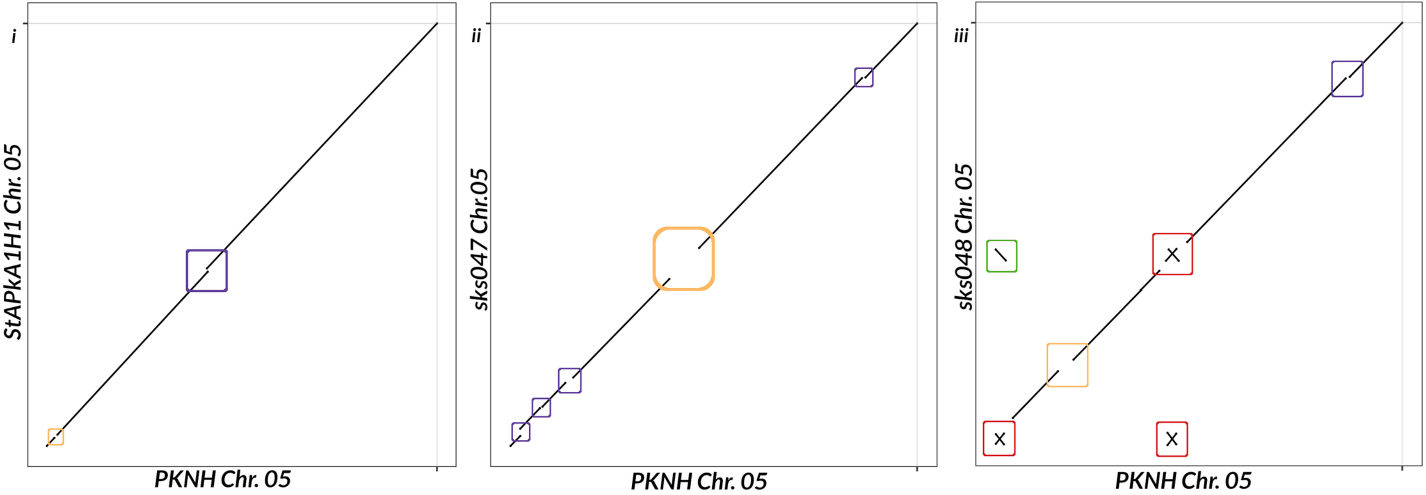

SI Fig 4A. Dot plots showing draft genomes aligned against the PKNH reference genome with minimum alignment 10kB. Chromosome 5 is given for StAPkA1H1 (i), sks047 (ii) and sks048 (iii), as an example where frameshifts are outlined in purple, gaps outlined in orange, inversions outlined in green and inverted repeats in red. Duplications are not shown.
